# In Situ Encapsulation of SnS_2_/MoS_2_ Heterojunctions by Amphiphilic Graphene for High‐Energy and Ultrastable Lithium‐Ion Anodes

**DOI:** 10.1002/advs.202405135

**Published:** 2024-07-25

**Authors:** Wenjun Yu, Baitao Cui, Jianming Han, ShaSha Zhu, Xinhao Xu, Junxin Tan, Qunjie Xu, Yulin Min, Yiting Peng, Haimei Liu, Yonggang Wang

**Affiliations:** ^1^ Shanghai Key Laboratory of Materials Protection and Advanced Materials in Electric Power Shanghai University of Electric Power Shanghai 200090 China; ^2^ Department of Chemistry and Shanghai Key Laboratory of Molecular Catalysis and Innovative Materials Institute of New Energy Fudan University Shanghai 200433 China

**Keywords:** anode materials, bimetallic sulfides, graphene, high‐energy, lithium‐ion batteries

## Abstract

Lithium‐ion batteries with transition metal sulfides (TMSs) anodes promise a high capacity, abundant resources, and environmental friendliness, yet they suffer from fast degradation and low Coulombic efficiency. Here, a heterostructured bimetallic TMS anode is fabricated by in situ encapsulating SnS_2_/MoS_2_ nanoparticles within an amphiphilic hollow double‐graphene sheet (DGS). The hierarchically porous DGS consists of inner hydrophilic graphene and outer hydrophobic graphene, which can accelerate electron/ion migration and strongly hold the integrity of alloy microparticles during expansion and/or shrinkage. Moreover, catalytic Mo converted from lithiated MoS_2_ can promote the reaction kinetics and suppress heterointerface passivation by forming a building‐in‐electric field, thereby enhancing the reversible conversion of Sn to SnS_2_. Consequently, the SnS_2_/MoS_2_/DGS anode with high gravimetric and high volumetric capacities achieves 200 cycles with a high initial Coulombic efficiency of >90%, as well as excellent low‐temperature performance. When the commercial Li(Ni_0.8_Co_0.1_Mn_0.1_)O_2_ (NCM811) cathode is paired with the prelithiated SnS_2_/MoS_2_/DGS anode, the full cells deliver high gravimetric and volumetric energy densities of 577 Wh kg^−1^ and 853 Wh L^−1^, respectively. This work highlights the significance of integrating spatial confinement and atomic heterointerface engineering to solve the shortcomings of conversion‐/alloying typed TMS‐based anodes to construct outstanding high‐energy LIBs.

## Graphical abstract

By encapsulating heterostructured SnS_2_/MoS_2_ nanoparticles in amphiphilic double‐layer‐graphene sheets, a hierarchical structure with a robust heterointerface is developed for lithium‐ion anodes with high volumetric and gravimetric capacities; this structure exhibited excellent electrochemical lithium‐ion storage performance, high catalytic reaction activity and fast electron/ion transport.

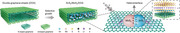

## 1 Introduction

Motivated by the rapid development of portable electronics, electric vehicles, and grid‐scale energy storage, advanced lithium‐ion batteries (LIBs) with high gravimetric and volumetric energy densities have attracted increasing amounts of attention.^[^ ^1^ ^]^ However, commercial graphite anodes, which account for ≈50% volume of a full cell, have nearly approached their capacity ceiling (theoretical gravimetric and volumetric capacities of 372 mAh g^−1^ and 841 mAh cm^−3^).^[^ ^2^ ^]^ Thus, the evolution of next‐generation anode materials with a high capacity, a small occupied volume, and good stability must be developed. Among the various anode candidates, transition metal sulfides (TMSs) with conversion‐alloying lithium storage mechanisms have attracted great interest because they provide a much higher theoretical volumetric capacity (*e.g*., 2562 mAh cm^−3^ for Sn‐based sulfides)^[^ ^3^ ^]^ than insertion‐type graphite anodes, and exhibit weak metal‐sulfur bonds and excellent redox reversibility. Based on their high capacity and low voltage plateau (< 0.5 V), SnS_2_‐based anodes are expected to achieve satisfactory utilization in high‐energy LIBs. Unfortunately, several inherent problems, such as poor conductivity, dramatic volume changes (260%),^[^ ^4^ ^]^ low initial Coulombic efficiency (ICE, <70%),^[^ ^5^ ^]^ and deteriorative stability, restrain their practical applications. The reasons for the low ICEs of TMS‐based anodes are associated with irreversible conversion reaction from lithiated TMSs to metals, SEI formation, active material loss during dramatic volume change, and Li‐ion trapping by detection sites.^[^ ^6^ ^]^

Hitherto, many efforts, such as TMSs nanoengineering,^[^ ^7^ ^]^ electrolyte optimizing,^[^ ^8^ ^]^ and multi‐components hybridization,^[^ ^9^ ^]^ *etc*., have been developed to improve the ICEs, but an effective volume constrained design strategy after the full alloy lithiation is lacking. The so‐formed cracks related to volume expansion and/or shrinkage can cause electrode pulverization and expose the highly reactive surface to the electrolyte, leading to a reformed growth of the solid electrolyte interphase (SEI) and electrolyte consumption. Moreover, the ruptured alloy impedes the reconversion back to TMSs, resulting in low ICE and rapid capacity decay. Thus, the endeavors to improve ICE and cycling CE are still in their infancy. Fortunately, constructing a binary or multiple TMS heterostructure anodes is considered a highly effective strategy to address this obstacle.^[^ ^10^ ^]^ Forming a building‐in‐electric field (BIEF) among the heterostructure components with different bandgaps can promote the reaction kinetics and accelerate electron transfer across the heterointerface, thereby maximizing the utilization of TMSs. Moreover, restacking and agglomeration of TMSs can be suppressed by the incorporation of multiple components, thus alleviating volumetric strain during repeated cycling.^[^ ^11^ ^]^ Benefiting from these merits, the SnS_2_/MoS_2_ heterostructure has been proven to improve the ICE and cycling stability, potentially increasing the capacity contribution of SnS_2_ due to the Mo existence.^[^ ^12^ ^]^ Nevertheless, brittle inorganic heterojunctions have difficulty resisting drastic volume expansion, and the SEI layer is mechanically unstable under the huge interfacial fluctuations and constant cycling reforms, leading to a low CE. Moreover, heterointerfaces with high resistance are rapidly passivated by repeated and nonsynchronous conversion/alloying‐dealloying processes,^[^ ^13^ ^]^ resulting in rapid capacity loss, structural collapse, and sluggish ion/electron kinetics.

To address these obstacles, incorporating heterojunction into bendable conductive agents (e.g., carbon nanofibers, carbon nanotubes, and reduced graphene oxide)^[^ ^14^ ^]^ to realize high ion/electron conductivity and high initial Coulombic efficiency is desirable. However, weak bonding between heterojunctions and carbonaceous scaffolds limits the formation of robust interphases, resulting in cracking or detachment of active materials from substrates during cycling and further triggering SEI reactions at the exposed surface.^[^ ^15^ ^]^ How to maintain the benefits of the SnS_2_/MoS_2_ heterostructure while achieving high ion/electron conductivity, high ICE/cycling CE, and sufficient mechanical strength remains challenging. In addition, in‐depth investigations of the relationship between the underlying reaction mechanisms and intermolecular interactions at the heterointerface and their effects on the electrochemical properties are worth further exploration.

**Figure 1:**
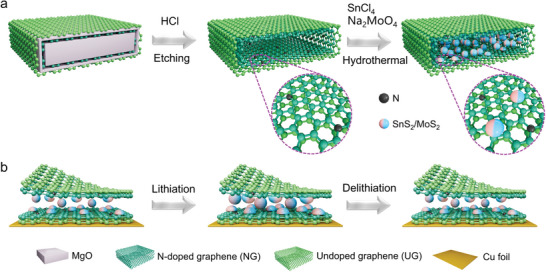
Schematic illustration of a) the synthesis of SnS_2_/MoS_2_/DGS composites by selective growth within amphiphilic double‐layered graphene, and b) their lithiation and delithiation processes, during which the composite electrode has both high conductivity and mechanical stability to resist large volume fluctuations of the SnS_2_/MoS_2_ nanoparticles, and thus maintain long‐term cycling.

To this end, we envision a design of synchronous integration of SnS_2_/MoS_2_ heterostructures with 2D hollow double‐graphene sheet (DGS) (denoted SnS_2_/MoS_2_/DGS). Specifically, the 2D confined space has a prominent advantage in terms of compatibility with interoverlapped superstructures of SnS_2_/MoS_2_ heterojunctions compared with 1D channels and 3D spaces.^[^ ^16^ ^]^ As illustrated in **Figure** **1**a, the DGS structure consists of an inner hydrophilic graphene sheet (nitrogen‐doped) and an outer hydrophobic graphene sheet (undoped), which were prepared by chemical vapor deposition (CVD) using acetonitrile and methane as precursors, respectively (Figure S1, Supporting Information), and subsequent removal of a catalytic MgO template to obtain a hollow DGS (the crystalline structure data and morphologies of the intermediate products are shown in Figures S2–S4, Supporting Information). The inner N‐doped graphene sheet provides relatively homogeneous temperature and concentration fields to efficiently control the nucleation and growth of the heterojunction,^[^ ^17^ ^]^ while the outer undoped graphene sheet with superior wettability to the organic electrolyte boosts Li‐ion transport over the heterointerface. Such biphilic confinement space and in situ encapsulation can effectively suppress stacking of SnS_2_/MoS_2_ in the *c*‐direction, it thus exhibits the morphology of nanoparticles or nanoclusters that shorten ion diffusion paths and improve the loading capacity, which is critical for high‐energy‐density LIBs. Furthermore, such an intriguing SnS_2_/MoS_2_/DGS integrated architecture is expected to control and cushion the large volume changes during the conversion/alloying‐dealloying process (Figure 1b), and to inhibit the restacking and agglomeration of nanoparticles. Through the elaborate design combining structural and heterointerfacial functions, the metallic heterointerface effectively reduces the electron/ion transport barriers and alleviates the mechanical stress upon cycling, significantly improving the power, energy output, and cycle life.

## 2 Results and Discussion

### 2.1 Materials Synthesis and Characterization

**Figure 2:**
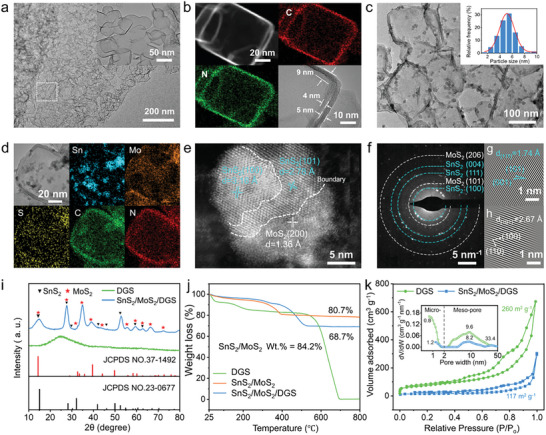
Morphology and structure of DGS and SnS_2_/MoS_2_/DGS composite. a) TEM images of DGS (the inset shows interconnected hollow subnanosheets), and its b) TEM‐EDX elemental maps of C and N. c) TEM images of the composite, and (inset) corresponding particle size distribution, and d) EDX elemental mapping of the composite. e) HRTEM image of a polycrystalline SnS_2_/MoS_2_ particle in the heterostructure composite. f) Corresponding SAED pattern with a collection of blue diffraction rings indexed to the (100), (111), and (004) planes of SnS_2_, and white rings assigned to the (101) and (206) planes of MoS_2_. g,h) Inverse fast Fourier transform (FFT) lattice images of (g) SnS_2_ (111), and (h) MoS_2_ (101). i) XRD patterns of DGS and the SnS_2_/MoS_2_/DGS composite. j) TGA curves in an air atmosphere at a heating rate of 10 °C min^−1^. k) N_2_ adsorption–desorption isotherms and corresponding pore size distributions (the inset of Figure 2k).

For demonstration, the scanning electron microscopy (SEM) images of DGS show a spherical and flower‐like structure composed of wrinkled nanoflakes with an approximate thickness of ≈160 nm and a corrugated rough surface after HCl treatment (Figure S5, Supporting Information). The inner (nitrogen‐doped) graphene of the DGS exhibits a contact angle change from 37° to 0° after 1 s, indicating a hydrophilic feature endowed by N‐doping (Figure S6, Supporting Information). Conversely, the outer (undoped) graphene maintains a large contact angle of ≈109° for 10 s, indicating a hydrophobic character. Such amphiphilic DGS can effectively capture sulfide particles within the inner layer and prevent their growth on the DGS surface. Moreover, the transmission electron microscopy (TEM) image (**Figure** **2**a), along with a higher‐magnification image, shows porous nanoflakes composed of many subnanosheets, with smaller sizes (≈30 ×   50 nm) interpenetrated with each other, resulting from fast pyrolysis of the MgO template during high‐temperature calcination (≈1000 °C).^[^ ^18^ ^]^ The detailed contour and elemental mapping of a hollow subnanosheet were further verified by TEM imaging in dark field mode (Figure 2b). The distance between the inner and outer layers (≈9 nm), and corresponding wall thicknesses of 4 and 5 nm (≈11–14 layers of graphene) can be observed. The two elements C and N are uniformly dispersed in the hollow DGS and enriched around high‐energy edge sites, indicating the successful introduction of nitrogen dopants.

After identifying the morphology and interfacial character of the hollow DGS, we studied the morphology, nanostructure, and composition of SnS_2_/MoS_2_/DGS. By in situ one‐step encapsulation, the corresponding morphology still presents a smooth and conformal morphology without nanoparticles attached on the outside of the DGS and remains nearly unchanged in thickness (≈160 nm, Figure S7, Supporting Information), verifying the confinement space of the DGS with the ability to accommodate high‐loading nanoparticles. Furthermore, the TEM images show that SnS_2_/MoS_2_ nanoparticles are fully encapsulated within the DGS (Figure 2c), with an average particle size of 5–6 nm, confirming the trapping ability of the inner layer to realize the selective growth of sulfide particles. The combined energy dispersive X‐ray (EDX) mapping reveals homogeneous spatial distributions of Sn, Mo, S, N, and C over the detected range of the composite materials (Figure 2d). High‐resolution TEM (HRTEM) further reveals a grain boundary of a SnS_2_ and MoS_2_ heterojunction, as marked by the dashed lines in a single ultrafine nanocrystal (Figure 2e). The phase boundaries generated by bimetallic sulfides greatly enhance the interfacial lithium‐ion transfer kinetics.^[^ ^19^ ^]^ The corresponding selected area electron diffraction (SAED) pattern (Figure 2f) further indicates the polycrystalline structure of SnS_2_/MoS_2_, while no diffraction ring of DGS is found due to its amorphous feature and more defects. Locally oriented lattice fringes with interplanar spacings of 1.74 and 2.67 Å are indexed to the (111), and (101) planes of SnS_2_ and MoS_2_ respectively (Figure 2g,h), confirming the successful construction of the SnS_2_/MoS_2_/DGS composite.

Consistently, Figure 2i shows the X‐ray diffraction (XRD) patterns of DGS and the SnS_2_/MoS_2_/DGS composite. The SnS_2_ and MoS_2_ patterns are assigned to the standard JCPDS No.23‐0677^[^ ^12^ ^]^ and JCPDS No.37‐1492,^[^ ^20^ ^]^ while the DGS exhibits the broad (002) peak at 26.8° corresponding to graphene (JCPDS No.41‐1487).^[^ ^21^ ^]^ The broadened peaks of the composites compared to those of individual SnS_2_ and MoS_2_ can be attributed to the nanosized domains generated along the basal plane,^[^ ^22^ ^]^ while no diffraction peak corresponding to the weakly crystalline DGS is observed in the XRD pattern. We also found that as‐synthesized composites via one‐step or two‐step iterative methods show similar diffraction peaks yet present distinct morphologies (Figures S7 and S8, Supporting Information). In comparison with the former, the latter displays apparent particle agglomeration on the outer edge of the DGS due to the prior space‐occupying of the first sulfide after a two‐step encapsulation. These findings verify that SnS_2_/MoS_2_ nanoparticles prefer to grow on a nitrogen‐doped graphene surface than the metal sulfide surface.

The chemical composition of the SnS_2_/MoS_2_/DGS composite was further determined using X‐ray photoelectron spectroscopy (XPS) (Figure S9, Supporting Information), where the deconvoluted peaks at binding energies of 284.2 eV (C 1*s* spectra), 400.3 eV (N 1*s*), 494.6/486.1 eV (Sn 3*d*_3/2_ *and Sn 3d*_5/2_), 231.5/228.4 eV (Mo 3*d*_3/2_ *and Mo 3d*_5/2_), and 162.5/161.2 eV (spin‐orbit couple S 2*p*_1/2_ *and* S 2*p*_3/2_) are assigned to C‐C/C‐N derived from the DGS, Sn‐S, and Mo‐S.^[^ ^23^ ^]^ These results were observed in many sulfide‐based heterojunctions and indicate the coexistence of Sn^4+^ and Mo^4+^.^[^ ^24^ ^]^ Several concurrent signals at 235.0 eV (Mo 3*d*), 225.4 eV (S 2*s* in MoS_2_), and 168.5 eV(S 2*p*) are attributed to Mo‐O arising from a partially oxidized surface,^[^ ^25^ ^]^ and Mo─S─Sn bonds.^[^ ^26^ ^]^ Moreover, the Sn 3*d* and S 2*p* peaks of the composite are both shifted to lower binding energies (≈−0.7 and −0.3 eV) compared with those of pristine SnS_2_, implying the transfer of electrons from Mo to Sn sites and an increase in the density of sulfur vacancies.^[^ ^27^ ^]^ The accurate constituents of the SnS_2_/MoS_2_/DGS composite were examined by thermogravimetric analysis (TGA) coupled with inductively coupled plasma atomic emission spectroscopy (ICP‐AES). The ICP‐AES results give an Sn/Mo ratio of 4.84 in the composite, which is nearly in line with the feed ratio of 5:1, whereby the weight fractions of SnS_2_, MoS_2_, and DGS in the composite are 74.1%, 13.4%, and 12.5%, respectively, based on TGA analysis (Figure 2j; Tables S1 and S2, Supporting Information).

Figure S10 (Supporting Information) compares the Raman spectra of DGS and SnS_2_/MoS_2_/DGS. The peaks at 320, 389, and 416 cm^−1^ are attributed to the vibrations related to the symmetric stretching of Sn‐S bonds (*A*_1*g*_), and the opposite stretching of Mo─S bonds in‐plane ($E_{2g}^{1})$ and out‐of‐plane (*A*_1*g*_),^[^ ^28^ ^]^ while the two broad peaks at 1328 and 1585 cm^−1^ represent the D (defect‐related) and G (graphitization degree) bands. The *I_D_/I_G_* intensity ratio of the composite is greater than that of DGS (1.52 vs 0.96), suggesting that the composite has more active sites for Li‐ions transfer and storage.^[^ ^29^ ^]^ Figure 2k further shows the N_2_ adsorption/desorption isotherms of DGS and SnS_2_/MoS_2_/DGS. Brunauer−Emmett−Teller (BET) analysis indicates that the specific surface area of DGS reaches 260 m^2^ g^−1^, and a high pore volume of 1.48 cm^3^ g^−1^ is calculated based on the Barrett−Joyner−Halenda (BJH) method. The composite exhibits a typical type‐IV isotherm with a hysteresis loop (0.4 < *P/P_0_* < 0.9) (surface area ≈ 118 m^2^ g^−1^ and pore volume of 0.35 cm^3^ g^−1^), indicating the existence of a hierarchical micro/mesoporous structure. An expanded micropore size from 0.8 to 1.2 nm and large mesopores of 33.4 nm are created by more defects within the porous DGS due to the nanoparticles encapsulation, facilitating rapid ion diffusion and mass transport.

### 2.2 Electronic Structure and Working Mechanism of Heterojunction Interface

**Figure 3:**
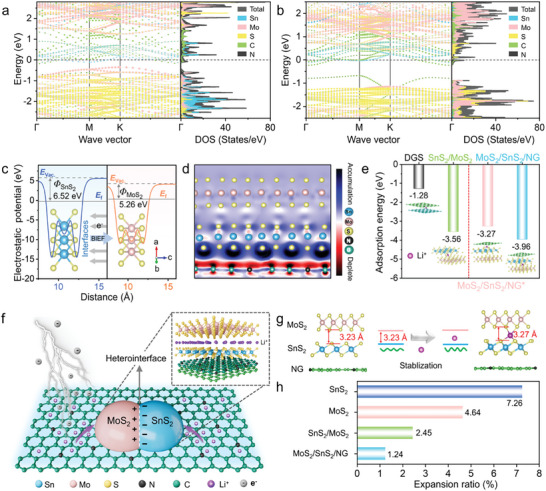
Band structures and DOS of a) MoS_2_/SnS_2_/NG and b) SnS_2_/MoS_2_/NG heterojunctions. c) Calculated work functions of SnS_2_ and MoS_2_. d) 2D electron density difference distribution of the MoS_2_/SnS_2_/NG heterostructure. The isosurface value is set to 0.0002 e/Bohr.^3^ e) Adsorption models and energies of a DGS, SnS_2_/MoS_2_, MoS_2_/SnS_2_/NG^*^, and MoS_2_/SnS_2_/NG heterojunctions. f) Scheme of the mechanism of the MoS_2_/SnS_2_/NG heterostructure anode for efficient lithium‐ion storage. g) Calculated atomic structures with the corresponding heterolayer‐expansion scheme after lithium‐ion intercalation. h) Comparison of the volume expansion ratios of the SnS_2_ and MoS_2_ interfaces, and the SnS_2_/MoS_2_ and MoS_2_/SnS_2_/NG heterointerfaces.

Density functional theory (DFT) calculations were performed to determine the electronic structure and vertical heterointerface of SnS_2_/MoS_2_/DGS at the atomic level. Considering that SnS_2_, MoS_2_, and DGS have different lattice parameters, the formed heterointerface can be divided into two models (Figure S11, Supporting Information), with a highly active SnS_2_ (001) or MoS_2_ (002) facet adjoining the N‐doped graphene (NG) in the DGS (denoted as MoS_2_/SnS_2_/NG and SnS_2_/MoS_2_/NG, respectively). **Figure** **3**a,b compares the energy band structures, total density of states (TDOS), and partial density of states (PDOS) of MoS_2_/SnS_2_/NG and SnS_2_/MoS_2_/NG. The band structures of the two composites both exhibit metallic features due to the existence of bands crossing at the Fermi level, leading to a higher conductivity than that of their counterparts based on the band structures, DOS, and integral DOS (Figures S12 and S13, Supporting Information). Compared with *n*‐type SnS_2_ (1.52 eV), MoS_2_ (1.28 eV), and SnS_2_/MoS_2_ (0.25 eV), the MoS_2_/SnS_2_/NG structure displays a continuous electron state in the DOS with a zero bandgap, thereby indicating accelerated interfacial charge carrier transport (Figure S14, Supporting Information).

The work function calculation (Figure 3c) further confirms that the electron flow in the heterojunction is self‐driven from MoS_2_ (5.26 eV) to SnS_2_ (6.52 eV), and from the outer undoped graphene (3.93 eV) to the inner N‐doped graphene (3.94 eV) (Figure S15, Supporting Information), indicating the formation of a BIEF that provides an additional electromotive force to facilitate electron conduction and self‐pumped Li‐ion transport through the heterointerface.^[^ ^30^ ^]^ To align with the prevailing heterointerface models that adopt SnS_2_ facets interconnected with various carbon substrates,^[^ ^31^ ^]^ we chose the MoS_2_/SnS_2_/NG heterostructure to investigate the inner phase‐junction interface through the charge density difference (Figure 3d), in which the red and blue regions represent depletion and accumulation of electrons. It is revealed that the electron distributions of the DGS and SnS_2_/MoS_2_ heterojunction are consistent with the DOS calculations (Figure S16, Supporting Information). Conversely, the MoS_2_/SnS_2_/NG shows a notable charge redistribution in which electrons transfer from MoS_2_ to SnS_2_ and homogenously distribute on the SnS_2_ side instead of locally accumulating (Figure 3d) due to the existence of the conductive DGS, confirming the existence of strong electronic coupling among the three components at the heterointerface.

Moreover, Figure 3e compares the adsorption energies and adsorption sites of Li^+^ ions, and the results indicate that the preferred lithium adsorption site lies at the MoS_2_/SnS_2_/NG heterointerface, where Li^+^ ions are intercalated between SnS_2_ and MoS_2_ or SnS_2_ and N‐doped graphene interlayers (denoted as MoS_2_/SnS_2_/NG and MoS_2_/SnS_2_/NG^*^, respectively). The adsorption energy of MoS_2_/SnS_2_/NG is markedly lower than that of SnS_2_/MoS_2_ (−3.96/−3.27 eV vs −3.56 eV) and the DGS (−1.28 eV) along the planar direction, demonstrating that this heterostructure can effectively reduce the ion diffusion barrier and achieve highly reversible Li‐ion insertion/extraction. The combination of electrophilicity and lithiophilicity endows the SnS_2_/MoS_2_/DGS heterointerface with low resistance and a strong anti‐polarization capability, as illustrated in Figure 3f. External electrons are transferred from the DGS to the binary metal sulfides; moreover, self‐driven internal electrons simultaneously jump between SnS_2_ and MoS_2_ via heterointerface until their Fermi level are balanced, leading to a high electronic conductivity ($\sigma_{e^{-}}$). Furthermore, the BIEF effectively attracts surrounding Li ions while allowing them to freely translocate within the electrolyte,^[^ ^32^ ^]^ leading to a high ionic conductivity ($\sigma_{Li^{+}}$). Such two electronic paths and fast Li‐ion migration in a synergistic manner greatly expand the electroactive microdomains and promote thermodynamic and kinetic stabilities.

In addition, the use of flexible DGS endows the SnS_2_/MoS_2_ heterojunction with superior structural stability upon Li‐ion intercalation. For the SnS_2_ or MoS_2_ homointerface, the layer expansion ratios after accommodating Li ions are 7.26% and 4.64%, respectively (Figure S17, Supporting Information), resulting in structural collapse with concomitant pulverization during cycling. For the SnS_2_/MoS_2_ heterointerface, the rigid stacked interface suppresses the interlayer spacing expansion (≈2.45%) from 3.27 to 3.35 Å (Figure 3g). Remarkably, the rigid‐flexible SnS_2_/MoS_2_/DGS heterointerface exhibits an ultralow layer expansion ratio of 1.24% (from 3.23 to 3.27 Å), which is far superior to that of SnS_2_, MoS_2_, and SnS_2_/MoS_2_ (7.26%, 4.64%, and 2.45%).

### 2.3 Electrochemical Performance of SnS_2_/MoS_2_/DGS Composite Electrode

**Figure 4:**
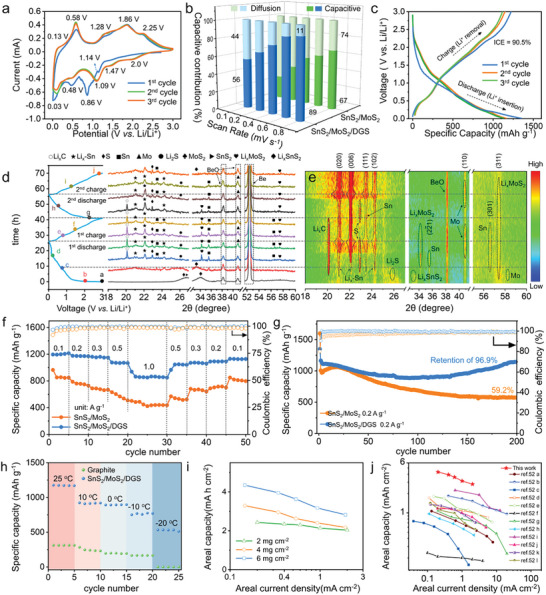
Electrochemical performance of SnS_2_/MoS_2_/DGS electrodes: a) CV curves of the first three cycles at a rate of 0.1 mV s^−1^. b) Comparison of the proportions of the capacitive and diffusive contributions. c) Galvanostatic discharge/charge profiles of the SnS_2_/MoS_2_/DGS electrode at 0.2 A g^−1^ during the first three cycles. d) In situ XRD at different discharge (D)/charge (C) depths (color online), and e) corresponding a contour plot during the initial two cycles. f) Rate performance and g) cycling performance at 0.2 A g^−1^. The active material loading is ≈2 mg cm^−2^. h) Discharged capacities of the SnS_2_/MoS_2_/DGS and graphite electrodes at 0.2 A g^−1^ and temperatures ranging from 25 to −20 °C. i) Areal capacity versus areal current density of SnS_2_/MoS_2_/DGS electrodes with mass loadings of 2, 4, and 6 mg cm^−2^. j) Comparison of the areal capacity of SnS_2_/MoS_2_/DGS anodes with that of representative anodes reported at different rates, including anodes from graphite, Sn‐based composites, heterojunction composites, and Si‐based composites.

The charge‐storage behavior of the SnS_2_/MoS_2_/DGS electrode was first characterized by cyclic voltammetry (CV) curves of the first three cycles at a 0.1 mV s^−1^ scan rate using coin‐type half cells (**Figure** **4**a). For the first discharge cycle, the cathodic peaks at 2.00–1.47, 1.14–0.48, and 0.1–0.03 V (vs Li/Li^+^) are ascribed to the initial Li insertion into SnS_2_ and MoS_2_, the conversion reaction of Li*_x_* SnS_2_ and Li*_x_* MoS_2_ to metallic Sn and Mo,^[^ ^33^, ^34^ ^]^ and the alloying reaction accompanied by Li insertion into DGS, as well as the formation of the SEI (≈0.86 V), respectively.^[^ ^12^ ^]^ In the subsequent two cycles, the reversible redox pair at 0.48/0.58 V is attributed to alloying/dealloying reaction of Sn,^[^ ^35^ ^]^ while the three redox pairs at 1.09/1.28, 1.47/1.86, and 2.00/2.25 V represent conversion reactions and Li insertion/extraction (Figure S18 and Table S3, Supporting Information),^[^ ^36^ ^]^ and all of these peaks overlap well and do not shift, implying excellent structural stability and more facile conversion kinetics. By comparison, the SnS_2_/MoS_2_ electrode shows a severe polarization on cycling, where apparent cathodic and anodic shifts toward lower and higher potentials respectively are observed (Figure S19, Supporting Information).

Moreover, CV curves at different scan rates were used to investigate the Li‐ion transport kinetics of the SnS_2_/MoS_2_/DGS electrodes (Figure S20, Supporting Information). The CV curves still exhibit well‐defined reversible redox peaks at higher scan rates, indicating a smaller polarization and a good rate capability. According to the equation for the peak current density (*i*) and scan rate (*ν*), *i* = *aν^b^*, whether the charge storage is a surface‐controlled capacitive (*k*_1_ *ν*) or diffusion‐controlled (*k*_2_ *ν*) process can be determined by the *b* value (where *k*_1_ and *k*_2_ are constants). A *b* value of 0.5 or 1 represents a diffusive or capacitive process, respectively, while a *b* value between 0.5 and 1 suggests coexisting charge mechanisms.^[^ ^37^ ^]^ The *b* values of 0.53 and 0.57 obtained for the composite electrodes by data fitting correspond to diffusion‐dominated conversion and alloy reaction, whereas the *b* values of 0.75 and 0.66 indicate capacitive‐dominated by lithiation intercalation. Compared with their bulk counterparts, the SnS_2_/MoS_2_/DGS electrodes provide 30%, 25%, 22%, 28%, and 22% improvements in the capacitive contribution at all C rates due to the reduced particle sizes and synergistic heterointerface (Figure 4b), confirming a fast pseudocapacitive response.

Consistently, Figure 4c shows the galvanostatic charge/discharge voltage profiles of the SnS_2_/MoS_2_/DGS electrode at 0.2 A g^−1^ for the first three cycles. The composite electrode shows a first discharge/charge capacity of 1350/1222 mAh g^−1^ and an ICE of 90.5%, which is far superior to the actual capacity of SnS_2_/MoS_2_ (1600/1070 mAh g^−1^), which has a lower CE of 66.8%, and those recently reported SnS_2_‐ or SnO_2_‐based anodes with low ICEs (<70%).^[^ ^38^ ^]^ The excess discharge capacity could be attributed to SEI formation and the irreversible Li‐ion insertion in DGS,^[^ ^21^ ^]^ The subsequent two cycles exhibit a slight capacity decay (only ≈3%), a low discharge plateau at 0.47 V, and a high CE of 99.3%, indicating a high full‐cell energy density. Noted that the capacities of the composite electrode are contributed by both the SnS_2_/MoS_2_ nanoparticles and DGS, the latter of which exhibits a capacity of 736, 693, 669, 639 and 508 mAh g^−1^ at the current density from 0.1 to 1.0 A g^−1^, respectively (Figure S21, Supporting Information). Considering the composites with 12.5 wt.% graphene contents, the capacity contributed by SnS_2_/MoS_2_ can be estimated as 1123, 1083, 1066, 987, and 787 mAh g^−1^, improving the utilization of bimetallic heterojunctions.

To investigate the structural evolution of the SnS_2_/MoS_2_/DGS electrode, in situ XRD was employed during the initial two cycles at 0.2 A g^−1^. Figure 4d shows that characteristic peaks of SnS_2_ and MoS_2_ can be identified at open‐circuit voltage (OCV), and the strong reflections at 38.3°/40.7°, and 45.4°/50.5°/52.4° are attributed to BeO and Be. As the first discharge proceeds, the diffraction peaks of binary sulfides at 27.6° and 34.1° almost disappear in sequence, while those of Li*_x_* SnS_2_ and Li*_x_* MoS_2_ emerge at 33.4° and 34.1°/57.5° (JCPDS: 22–0692 and 44–1078). Furthermore, the diffraction signals of metallic Sn, Mo, and, S and Li_2_S species appear at 23.7°, 40.8°, 22.8°, and 25.9° (JCPDS: 87‐0794, 42‐1120, 13‐0144, and 04‐2752) respectively, corresponding to the (111), (110), (101), and (111) planes. With further lithiation, Sn peaks gradually decrease while the peaks of Li*_x_* Sn at 21.4°, 22.3°, and 23.4° strengthen, accompanied by Li intercalation into DGS at 20.4°.^[^ ^39^ ^]^ Following the Li‐ion extraction, the signals of the alloy become weaker and the breaking of Li─S bonds leads to the reappearance of Sn metal; however, no obvious peaks for Li*_x_* SnS_2_ are observed due to its amorphous nature and/or small crystallite size.^[^ ^40^ ^]^ The second discharge/charge process exhibits similar results but proceeds in an almost reversible fashion, suggesting good reversibility. The corresponding 2D contour plot shows that such diffraction peaks associated with Li‐ion intercalation, conversion, and the subsequent alloying reaction can be used to trace the crystal structure evolution (Figure 4e), and the results confirm that the capacity contribution not only depends on the alloying reaction but also arises from the conversion reaction.

Figure 4f compares the rate capability of SnS_2_/MoS_2_/DGS electrodes at various current densities of 0.1, 0.2, 0.3, 0.5, and 1.0 A g^−1^, at which the electrodes deliver specific capacities of 1215, 1170, 1150, 1067, and 851 mAh g^−1^, respectively. When the cycling current density is returned to 0.1 A g^−1^, the capacity slightly decreases and remains at 1129 mAh g^−1^ after 50 cycles due to the partial irreversibility of the structural evolution. The capacities of composite electrodes with higher ICE are much greater than that of SnS_2_/MoS_2_ electrodes at all current densities. Moreover, Figure S22 (Supporting Information) shows the Li‐ion solid‐state diffusion within two cycles at 0.2 A g^−1^ obtained using the galvanostatic intermittent titration technique (GITT). The calculated diffusion coefficients of Li ions ($D_{Li^{+}}$) in the SnS_2_/MoS_2_/DGS electrode range from 10^−6^ to 10^−4^ cm^2^ s^−1^, which are four to five orders of magnitude greater than those of reported metal sulfide‐and SiO*_x_*‐based composite electrodes (10^−10^ to 10^−9^ cm^2^ s^−1^).^[^ ^41^, ^42^, ^43^ ^]^ Such a higher diffusion coefficient of the composite electrode is strongly associated with the functional structural components (ultrafine nanoparticles and favorable hollow DGS nanosheets), BIEF establishment, and low‐resistance heterointerface integration, which lead to shorter diffusion paths and faster Li diffusion dynamics.

Figure 4g further displays the cycling stability of the SnS_2_/MoS_2_/DGS and SnS_2_/MoS_2_ electrodes at 0.2 A g^−1^, where the capacity delivered by both electrodes continuously increases from the initial capacity due to electrode activation and a stable SEI contribution. The SnS_2_/MoS_2_/DGS electrode delivers discharge capacities of 1164 and 1128 mAh g^−1^ in the 2nd and 200th cycles, respectively, with 96.9% capacity retention, outperforming the SnS_2_/MoS_2_ electrode, which delivers specific capacities of 970 and 575 mAh g^−1^ in the 2nd and 200th cycles, respectively, with 59.2% capacity retention. In terms of the CE, the SnS_2_/MoS_2_/DGS electrode provides a higher efficiency of 90.5% and 99.9% than that of its counterparts (66.8% and 99.0%) in the 1st and 200th cycles, respectively (Figure S23, Supporting Information), suggesting that it will have an improved cycle life in full cells. In addition, we also observed that the SnS_2_/MoS_2_/DGS electrode shows an obvious capacity fluctuation during the 116th cycle, where the enhanced capacity is attributed to the catalytic activation of MoS_2_. MoS_2_ has been reported to play the role of a catalyst that facilitates the decomposition of Li_2_S and improves the reversibility of sulfur,^[^ ^12^, ^44^, ^45^, ^46^, ^47^, ^48^ ^]^ which enhances the conversion capacity contribution of Sn in heterojunction. Some lattice defects were generated during the cycling process, which successfully unlocked the basal planes of binary sulfides to provide extra ion diffusion channels and storage sites.^[^ ^49^ ^]^ Besides that, SnS_2_/MoS_2_ nanoparticles are shrinking on cycling, generating smaller particles that can deliver higher capacities and still be encapsulated in the robust and conductive DGS scaffold.^[^ ^50^ ^]^ In contrast, such a phenomenon is not observed in pure heterojunction electrodes because unconstrained sulfide nanoparticles easily disassemble from conductive carbon due to the dramatic volume expansion/extraction during cycling, resulting in catalyst deactivation. Therefore, the ability of the SnS_2_/MoS_2_/DGS composite to significantly improve the rate capability and capacity, CE, catalytic efficiency, and cycle life under deep‐cycling conditions is of particular importance for high‐energy LIB applications.

Post‐cycling impedance analysis indicates that the cycled SnS_2_/MoS_2_/DGS electrode has a lower solution resistance (R_s_) of 4 Ω, a much lower charge‐transfer heterointerface resistance (R_ct_) of 37 Ω, a lower contact resistance (R_f_) of 84 Ω, and a lower Li‐ion diffusion resistance (Z_w_) of 86 Ω (Figure S24 and Table S4, Supporting Information) than the SnS_2_/MoS_2_ electrode after cycling (vs 7, 78, 125, and 572 Ω, respectively), confirming the coupling effects of the dual electronic paths from both the DGS to SnS_2_/MoS_2_ and within the heterojunction itself. Cross‐sectional SEM was also performed on the fresh SnS_2_/MoS_2_/DGS electrode and the electrode after 200 cycles (Figure S25, Supporting Information), in which all electrodes were peeled off from the Cu foil to prevent edge curling from influencing the observation. Under the same active material loading conditions (≈2 mg cm^−2^), the thickness of the cycled composite electrode slightly increases by 13.9% (from 45.3 to 56.1 µm), while the SnS_2_/MoS_2_ electrode exhibits a huge expansion of 101.4% (from 29.5 to 59.4 µm). Moreover, no significant cracks or delamination are observed in the SnS_2_/MoS_2_/DGS electrode, indicating that the electrode structure is highly stable during the repeated cycling process. Figure S26 (Supporting Information) further confirms that the SnS_2_/MoS_2_ nanoparticles are still confined within the hollow DGS after 200 cycles; thus, detachment of the active materials from the hollow DGS has been avoided, and the mechanical and electrical connections across the heterointerfaces are maintained. We also characterized the cycled composite electrodes using XPS (Figure S27, Supporting Information) to identify the phase transition of fully discharged state, and found that the XPS peaks have no obvious shift, indicating complete reconversion of Sn with Li_2_S. Therefore, these post‐cycling evaluations demonstrate that the morphology and chemical composition of the SnS_2_/MoS_2_/DGS electrodes possess long‐term stability.

To further investigate the low‐temperature performance (from 25 to −20 °C), all tested electrodes were subjected to a cycling protocol consisting of two cycles at 25 °C and 0.1 A g^−1^ and a subsequent cycle at 10/0/−10/−20 °C and 0.1, 0.2, 0.3, 0.5, or 1 A g^−1^. Figure 4h compares the galvanostatic discharge capacities of the SnS_2_/MoS_2_/DGS and graphite electrodes at different temperatures at a constant 0.2 A g^−1^. The SnS_2_/MoS_2_/DGS electrodes deliver higher capacity retentions of 78.5%, 77.0%, and 66.2% than the graphite electrodes (74.3%, 62.5%, and 53.2%) at 10, 0, and −10 °C, respectively, with a well‐profiled voltage plateau at −10 °C and 1.0 A g^−1^ (Figure S28, Supporting Information). Even at −20 °C, the composite electrode still provides a capacity of 512 mAh g^−1^ at 0.2 A g^−1^, whereas the graphite electrodes hardly work due to severe polarization.^[^ ^51^ ^]^

Considering realistic industrial applications, Figure 4i shows the rate performance of SnS_2_/MoS_2_/DGS electrodes with higher mass loadings (active material only) at different current densities. As the areal current density increases, the areal capacities maintain a similar declining trend due to nonnegligible diffusion limitations. The composite electrode with a mass loading of 6 mg cm^−2^ delivers areal capacities of 4.3, 3.9, 3.6, 3.1, and 2.8 mAh cm^−2^ at areal current densities of 0.2, 0.4, 0.6, 1, and 2 mA cm^−2^ and still retains 64.8% of its capacity at 2.0 mA cm^−2^, thereby maximizing the utilization and packing density of composite electrodes. To fully compare representative high‐capacity anodes for LIBs with the prior art, Figure 4j plots the areal capacity at different areal current densities of SnS_2_/MoS_2_/DGS and other electrodes, including electrodes consisting of commercial graphite, graphene, Li_4_Ti_5_O_12_ (LTO), Sn‐based composites, heterojunction composites(such as carbonous heterostructure composites), and Si‐based composites (Table S5, Supporting Information).^[^ ^52^ ^]^ Compared with low‐capacity graphite, high‐capacity Sn‐based composites, and Si‐based composites, the SnS_2_/MoS_2_/DGS electrode with high loading (≈6 mg cm^−2^) well exceeds the abovementioned anodes at all areal current densities, delivering a higher areal capacity of over 2.8–4.3 mAh cm^−2^ than those of other anodes of 0.2–2.2 mAh cm^−2^ in a current density range of 0.1–2.0 mA cm^−2^.^[^ ^5^, ^53^, ^54^ ^]^ All the above evidence collectively corroborates that the as‐prepared SnS_2_/MoS_2_/DGS heterostructure provides a robust architecture to address the issue of the high‐capacity TMS could suffering from low ICE and performance deterioration under extreme conditions.

### 2.4 Structural Stability and Electrochemical Performance of the Full Cell

**Figure 5:**
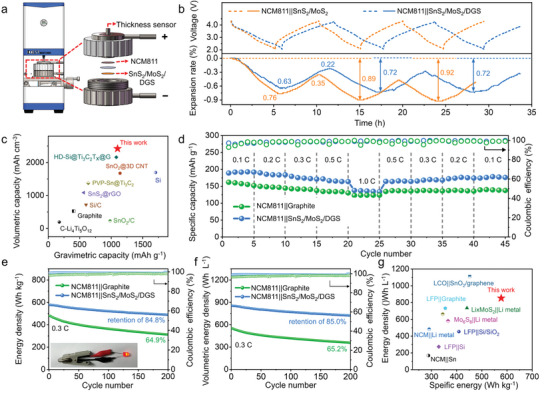
Electrochemical performance of full cells. a) Schematic of the experimental apparatus with a cell mold used for in situ thickness change monitoring. b) Comparison of the voltage‐dependent thickness variations of the SnS_2_/MoS_2_/DGS and SnS_2_/MoS_2_ electrodes during the first three cycles. c) Comparison of the specific volumetric capacity of the SnS_2_/MoS_2_/DGS electrode (active materials only) with that of reported anode materials, including graphite, LTO, Sn/C, and Si/C. d) Rate performance of NCM811||SnS_2_/MoS_2_/DGS cells at different C‐rates. e) Gravimetric and f) volumetric energy densities of NCM811||SnS_2_/MoS_2_/DGS cells and NCM811||graphite cells. g) Projected specific energy and energy density of various Li‐based batteries, including those with graphite, LTO, Mo_6_S_8_‐, Li‐, Sn‐, and Si‐based anodes.

To address this obstacle, we adopted an in situ mechano‐electrochemical technique to examine the thickness expansion of the SnS_2_/MoS_2_/DGS anode in a full‐cell assembly with a stable Li[Ni_0.8_Co_0.1_Mn_0.1_]O_2_ (NCM811) cathode with a bulk density of 4.5 g cm^−3^, which has a negligible volume change (<5%).^[^ ^55^ ^]^ The experimental apparatus with a cell mold is illustrated in **Figure** **5**a, in which the force spring and thickness sensor are assembled in the expansion analysis system. The mass loadings of the well‐dried NCM811 and SnS_2_/MoS_2_/DGS electrodes were controlled at 9.5 and ≈2 mg cm^−2^, respectively, corresponding to a negative/positive (N/P) ratio of 1.1 (Figure S29, Supporting Information). For comparison, reference cells were also assembled using the SnS_2_/MoS_2_ anode, and all tested full cells were operated in a voltage range of 2.0–4.25 V at 0.3 C (1C = 188 mA g^−1^). Figure S30 (Supporting Information) shows that the SnS_2_/MoS_2_/DGS electrode exhibits a very low thickness expansion of 0.81% during the prelithiation process, so the thicknesses in the un‐lithiated/fully‐ lithiated states can be obtained. When this fully charged anode was then assembled into a full cell, the full cell with the SnS_2_/MoS_2_/DGS electrode exhibited a lower thickness contraction than that of the full cell with the SnS_2_/MoS_2_ electrode (0.63% vs 0.76%) during the first discharge process (Figure 5b), especially the former, delivered a higher capacity. The reverse‐charging process shows that the thickness expansion of the SnS_2_/MoS_2_/DGS electrode is closer to the fully‐lithiated state compared with the delithiated thickness of the SnS_2_/MoS_2_ electrode, demonstrating the excellent structural stability of the SnS_2_/MoS_2_/DGS electrode. In the subsequent two cycles, the thickness variations of the composite electrode are unchanged, while the thickness fluctuation of the SnS_2_/MoS_2_ electrode appreciably increases in the third cycle, suggesting that the robust DGS significantly inhibits the volume expansion of SnS_2_/MoS_2_.

Encouraged by the superior structural integrity, the volumetric capacities versus gravimetric capacities of representative electrodes (total mass of electrode materials) in an unlithiated state are compared with those of the SnS_2_/MoS_2_/DGS electrode in Figure 5c (Table S6, Supporting Information).^[^ ^56^ ^]^ The SnS_2_/MoS_2_/DGS electrode has a tap density of ≈2.20 g cm^−3^ and specific capacities of 1100 and 660 mAh g^−1^ at 0.2 and 1 A g^−1^, corresponding to volumetric capacities of 2420 and 1452 mAh cm^−3^, respectively. To be accurate and meaningful in presenting the volumetric capacity of the SnS_2_/MoS_2_/DGS anode, that of the thicker SnS_2_/MoS_2_/DGS electrode in the fully lithiated state was obtained, in which the electrode retained high volumetric capacities of 1210 and 726 mAh cm^−3^ at 0.2 and 1 A g^−1^, respectively, well outperforming the most recently reported anodes even though its gravimetric capacity is lower than that of porous Si (1697 mAh cm^−3^ at 0.2 A g^−1^).^[^ ^57^ ^]^

Benefiting from these merits of the SnS_2_/MoS_2_/DGS electrode in a half‐cell, we further investigated its electrochemical efficacy in full cells, including its rate performance, energy output, and cycle life, by projecting the electrochemical performance of full‐coin cells (see Table S7, Supporting Information for calculation parameters). Figure 5d compares the rate performance of the NCM811||SnS_2_/MoS_2_/DGS and NCM811||graphite cells (denoted as the modulated cell and reference cell, respectively) at different C‐rates, where the modulated cell delivers 191.8, 183.9, 171.2, 163.8, and 134.2 mAh g^−1^ at 0.1 C, 0.2 C, 0.3 C, 0.5 C, and 1 C, well surpassing the referenced cell at all C‐rates. Moreover, the average CEs of the cells with composite anodes are much higher (96.9%, 98.1%, 98.5%, 98.7%, and 99.1%) than that of the referenced cells, exhibiting high “lithium activity” even at elevated charge rates.^[^ ^58^ ^]^ Note that the specific capacity of the modulated cell recovers to 176.1 mAh g^−1^ when the rate returns to 0.1 C, reflecting the outstanding rate capacity. Moreover, Figure 5e shows the cycling performance of the modulated cell at 0.3 C, which provides an initial gravimetric energy density of 577 Wh kg^−1^ with a high‐power density of 173 W kg^−1^ (including the cathode, unlithiated anode, separator, and current collector), which is 1.2 times higher than that of the reference cell (480 Wh kg^−1^). As expected, the modulated cell still maintains a stable energy output of 490 Wh kg^−1^ with an energy retention of 85.0% after 200 cycles. The initial/average CE is 98.9/99.9% and 96.5/99.1% for the modulated cell and reference cell during cycling, respectively, further demonstrating the great potential of the former for practical high‐energy LIB applications.

Aided by DGS buffering‐release of stress during repeated cycles, the modulated cell also has a high volumetric energy density of 853 Wh L^−1^ with a total thickness of 129 µm (including the cathode, unlithiated anode, separator, and current collector), and this density is ≈1.6 times greater than that of the reference cell (551 Wh L^−1^) (Figure 5f; Figure S31, Supporting Information). Furthermore, Figure 5g provides a rough comparison of NCM811||SnS_2_/MoS_2_/DGS, LiFePO_4_(LFP)||graphite, LFP||LTO/SnO_2_/LTO, Mo_6_S_8_||Li, and other reported Sn‐ and Si‐based full cells^[^ ^59^ ^]^ according to the specific energy and volumetric energy density, which were estimated based on similar cell models. Notably, a LIB consisting of a high‐capacity SnS_2_/MoS_2_/DGS anode and a high‐voltage NCM811 cathode can deliver much higher volumetric energy density of 847 Wh L^−1^ than a Li ion‐based full cell when a 130‐µm‐thick anode in the fully lithiated state is adopted. This LIB is superior to most recently reported full cells and successfully lights up a small light‐emitting diode (LED) bulb (inset of Figure 5e). This excellent performance can be attributed to the robust architecture, active material loading, areal capacity, and N/P ratio and reveals that our high‐energy LIB based on the SnS_2_/MoS_2_/DGS design represents an important advance for future consumer electronics and electric vehicle applications.

## 3 Conclusion

In summary, The SnS_2_/MoS_2_/DGS heterostructure was successfully fabricated via in situ one‐step encapsulation. Combining high electron/ion conductivity with the ability to form robust heterointerfaces that have low resistance and anti‐concentration‐polarization capability, such composite electrodes effectively accommodate the large volume change of the alloy electrode and improve ICE (>90%). Through in situ characterizations coupled with DFT calculations, the interoverlapped structure between SnS_2_, MoS_2_, and N‐doped graphene can provide more lithium‐ion storage sites, accelerate electron/ion migration, and promote interfacial reaction kinetics, thereby significantly improving the reversibility of Sn to SnS_2_. Benefiting from these merits, the SnS_2_/MoS_2_/DGS electrodes deliver a high capacity of 1128 mAh g^−1^ for over 200 cycles with retention of 96.9%, and a high areal capacity of 4.3 mAh cm^−2^. Furthermore, when paired with a high‐voltage NCM811 cathode, the full cells with high energy density (577 Wh kg^−1^ or 847 Wh L^−1^) are achieved under the adoption of full‐lithiated thickness condition. With the deeper exploration of electrode materials in fundamental research and benchmarking with LIBs in the current market, this heterostructure anode can soon be expected to enable the construction of high‐energy‐density LIBs.

## Conflict of Interest

The authors declare no conflict of interest.

## Supporting information

Supporting Information

## Data Availability

Research data are not shared.
