# Supplementary material for: In Situ Encapsulation of SnS2/MoS2 Heterojunctions by Amphiphilic Graphene for High‐Energy and Ultrastable Lithium‐Ion Anodes
Source: Adv Sci (Weinh). 2024 Jul 25;11(36):2405135. doi: 10.1002/advs.202405135 (PMC11423093; doi:10.1002/advs.202405135)
Supplement: Supplementary file 1 — Supporting Information [file ADVS-11-2405135-s001.docx]

**Supporting Information**

***In Situ* Encapsulation of SnS_2_/MoS_2_ Heterojunctions by Amphiphilic Graphene for High-Energy and Ultrastable Lithium-ion Anodes**

*Wenjun Yu, Baitao Cui, Jianming Han, ShaSha Zhu, Xinhao Xu, Junxin Tan, Qunjie Xu, Yulin Min, Yiting Peng*, Haimei, Liu*, Yonggang Wang**

W.Yu, B. Cui, J. Han, S. Zhu, X. Xu, J. Tan, Q. Xu, Y. Min, Prof. Y. Peng, Prof. H. Liu

Shanghai Key Laboratory of Materials Protection and Advanced Materials in Electric Power, Shanghai University of Electric Power, Shanghai, 200090, China

E-mail: [pyt_1108@shiep.edu.cn](mailto:pyt_1108@shiep.edu.cn); [liuhm@shiep.edu.cn](mailto:liuhm@shiep.edu.cn)

Prof. Y. Wang

Department of Chemistry and Shanghai Key Laboratory of Molecular Catalysis and Innovative Materials, Institute of New Energy

Fudan University, Shanghai 200433, China.

E-mail: [ygwang@fudan.edu.cn](mailto:ygwang@fudan.edu.cn)

*Corresponding authors. Email:

[pyt_1108@shiep.edu.cn](mailto:pyt_1108@shiep.edu.cn); [liuhm@shiep.edu.cn](mailto:liuhm@shiep.edu.cn), and [ygwang@fudan.edu.cn](mailto:ygwang@fudan.edu.cn)

**Experimental Procedures**

**Synthesis of DGS:** The below-mentioned chemicals were purchased from Sigma-Aldrich and used as received. First, the Mg_5_(CO_3_)_4_(OH)_2_ template was synthesized according to a previously reported one-pot hydrothermal method.^[1]^ Briefly, 0.71 g magnesium nitrate hexahydrate (Mg(NO_3_)_2_·6H_2_O) and 0.79 g ammonium bicarbonate (NH_4_HCO_3_) were dissolved in 100 mL deionized water and vigorously stirred for 1 h. Then, the mixtures were transferred to a 100 mL Teflon-lined autoclave and heated to 120 °C for 6 h. The obtained white precipitate was washed and filtered by deionized water for several times, and dried overnight at 80 °C, followed by calcination at 600 °C for 6 h in air to obtain the MgO template.

Subsequently, chemical vapor deposition (CVD) was conducted by placing the MgO template in a quartz boat in a horizontal hot-walled tube furnace. For the synthesis of nitrogen-doped graphene (NG) on MgO, the furnace was heated to 900 °C in an Ar/H_2_ mixture gas, in which the gas flow rates were 400 mL min^−1^ and 200 mL min^−1^. Then another Ar stream (400 mL min^−1^) passing through an acetonitrile-filled flask also flowed into the furnace for 8 min to obtain the NG-MgO products. Once again, the as-synthesized NG-MgO was dispersed in 100 mL deionized water containing 0.71 g Mg(NO_3_)_2_·6H_2_O and 0.79 g NH_4_HCO_3_ and sonicated for 30 min. The mixture was subjected to hydrothermal synthesis again, washed, and dried at 80 °C overnight. The as-formed product was then coated with undoped graphene using CH_4_ (400 mL min^−1^) as the carbon source at 1000 °C for 5 min. Finally, the obtained samples were soaked in HCl (1 M) solution to remove the templates and obtain a double-graphene sheet (DGS).

**Synthesis of** **SnS_2_/MoS_2_/DGS, MoS_2_/DGS/SnS_2,_ and SnS_2_/DGS/MoS_2_ composites:**

The MoS_2_/SnS_2_/DGS composite was synthesized through a one-step hydrothermal reaction. The obtained DGS (0.27 g) were dispersed in a mixed solution containing SnCl_4_·5H_2_O (0.80 g), Na_2_MoO_4_·2H_2_O (0.14 g), and 0.69 g thioacetamide (TAA) and stirred for 8 h. Then the resulting suspension was transferred to a 100 mL Teflon-lined autoclave and heated to 200 °C for 16 h. The obtained samples were washed and dried at 80 °C for 12 h, followed by calcination at 450 °C for 30 min in Ar (200 mL min^−1^) gas to obtain the final products. The control SnS_2_/MoS_2_ sample was also prepared following similar procedures without DGS.

For comparison, we also adopted a two-step iterative method to synthesize SnS_2_/DGS/MoS_2_ and MoS_2_/DGS/SnS_2_ based on procedures similar to those used for SnS_2_/MoS_2_/DGS, except that onlySnCl_4_·5H_2_O or Na_2_MoO_4_·2H_2_O primarily reacted with the DGSs *via* hydrothermal treatment, and subsequently reacted with as-synthesized MoS_2_/DGS or SnS_2_/DGS by exchanging the precursor to obtain SnS_2_/DGS/MoS_2_ and MoS_2_/DGS/SnS_2_ composites, respectively. The collected products were washed, dried under vacuum at 80 °C, and calcined through the same procedures.

**Material Characterization:** X-ray diffraction (XRD) was performed using a Bruker D8 Advance diffractometer (Bruker., Germany) (Cu-Kα, λ = 1.5406 Å, scan rate of 5° min^-1^). Scanning electron microscopy (SEM) was conducted on a GeminiSEM 300 (ZEISS Ltd., Germany). Transmission electron microscopy (TEM) and high-resolution TEM (HRTEM) coupled with selected area electron diffraction (SAED) were performed with a JEM-2100F (JEOL Ltd., Japan) operating at 120 kV and a Titan Themis G2 60-300 (Thermo Fisher., America) operating at 300 kV. High-angle annular dark-field (HAADF) scanning transmission electron microscopy (STEM) equipped with energy-dispersive X-ray spectroscopy (EDS) was conducted to analyze the compositional distribution. The contact angle was measured using a JC2000DS2 instrument (Shanghai Zhongchen, China) equipped with a motion image capture camera. The composition of the composites was determined by thermogravimetric analysis (TGA, HITACHI STA200). Nitrogen sorption isotherms were obtained at 77 K with a Micromeritics ASAP 2460 system. The specific surface area was calculated by the Brunauer−Emmett−Teller (BET) method using an adsorption branch in a relative pressure range from 0.05 to 0.35. The pore size distribution was determined using the Non-localized Density Functional Theory (NLDFT) model. Raman spectra were obtained with a 532 nm excitation wavelength (Raman JY HR800 Spectrometer, France), and X-ray photoelectron spectroscopy (XPS, Thermo Scientific K-Alpha spectrometer, America) was performed. All collected XPS spectra were calibrated by the C1s spectral component at 285.0 eV and then fitted based on Gaussian-Lorentzian functions to perform peak deconvolution. Elemental ratios analysis was performed using inductively coupled plasma-atomic emission spectroscopy (ICP−AES, Agilent 5110, America).

**Density functional theory (DFT) calculations**: All DFT calculations were conducted by using the Vienna *ab* *initio* simulation package (VASP) with the projector augmented-wave (PAW) method used to describe the core-valence interactions.^[2]^ The Perdew−Burke−Ernzerhof (PBE) pseudopotentials of the generalized gradient approximation (GGA) functional^[3]^ were used to calculate the exchange−correlation interactions. Moreover, the Hubbard U correction of the strongly correlated system of d electrons in transition metal elements was considered (U_Sn_ = 1.5 eV, U_Mo_ = 3.2 eV).^[4]^ The van der Waals (vdw) interactions were corrected with the Grimme scheme (vdw-D3). The cutoff energy for the plane-wave basis was set to 400 eV, and convergence thresholds of 1.0 × 10^-5^ eV in energy and 0.02 eV Å^-1^ in force were used. For calculation of the band structures, the Brillouin zone (periodic boundary conditions) was sampled by a 3 × 3 × 1 *k*-point mesh with the Monkhorst−Pack scheme to optimize the geometric structures and density of states (DOS).^[5]^ In addition, spin polarization was considered in all calculations, using Gaussian broadening for the Fermi level and an unfolded width of 0.05 eV.

**Structure model and work function calculation:** The original models of N-doped graphene (NG), DGSs, SnS_2_, MoS_2_, and the SnS_2_/MoS_2_, SnS_2_/MoS_2_/NG, and MoS_2_/SnS_2_/NG heterostructures were constructed, where the DGS model was composed of a single-layer NG 5 × 5 supercell with four carbon atoms, substituted and a single-layer undoped graphene 5 × 5 supercell. Moreover, a single-layer SnS_2_ 3 × 3 supercell and a MoS_2_ 4 × 4 supercell were also modeled as references. The SnS_2_/MoS_2_/NG or MoS_2_/SnS_2_/NG heterointerfaces were alternately built from the single-layer SnS_2_ (001), MoS_2_ (002), and NG models. A 15 Å vacuum slab was set to prevent periodic interactions between neighboring layers in the *c-direction*. We further chose the work function ($W_{f}$) to calculate the minimal amount of energy needed to trap an electron from the Fermi level (*E_f_*) according to the difference between the vacuum level and the Fermi level.^[6]^ The resulting Δ*Φ* provides a meaningful measurable observable for characterizing homo-/hetero-interfaces in terms of electronic properties and the resulting energy driving to electron transfer.

**Adsorption energy and expansion ratio calculation**:

To compare Li-ion adsorption on different sites in the heterostructures, we calculated the adsorption energy (Δ*E*_ad_) according to the following equation:

|  | $\Delta E_{ad}=\Delta E_{total}-\Delta E_{bare}-\Delta E_{Li}$ | (1) |
| --- | --- | --- |

where Δ*E*_total_ is the total energy of the Li atom adsorbed on the anode, Δ*E*_bare_ is the energy of the unlithiated anode, and Δ*E*_Li_ is the energy of an isolated lithium atom. The expansion ratio (β) was theoretically calculated by dividing the original volume (*V*_0_) of anode active materials by the volume change (Δ*V*) after Li-ion accommodation:

|  | $\beta=\Delta V/V_{0}$ | (2) |
| --- | --- | --- |

**Electrochemical characterization:**

The synthesized sample (active materials), polyvinylidene fluoride (PVDF), and carbon black with an active material-to-carbon-to-binder mass ratio of 8:1:1 were mixed in N-methyl pyrrolidone (NMP) solvent to form a homogeneous slurry, which was uniformly cast on copper foil (9 μm, Canrd Co., Ltd) and dried at 80 ℃ overnight in a vacuum oven. Subsequently, the dried electrode was cut into disks with a diameter of 12 mm, and the average mass loading of the active materials was approximately 2.0 mg cm^-2^. CR-2032 coin cells were assembled in an argon-filled glove box (O_2_ < 0.01ppm, H_2_O < 0.01 ppm) by using as-prepared electrodes as the working electrode, a lithium foil Li disc (1.0 mm thick, 16.0 mm diameter, Canrd Co.,Ltd.) as the counter electrode, a PP-based membrane (Celgard 2500) as the separator, and 1 M LiPF_6_ in 1:1 v/v ethylene carbonate (EC) and diethyl carbonate (DEC, Sigma Aldrich) as the electrolyte. For the full cells, the SnS_2_/MoS_2_/DGS electrode was activated by a discharge lithiation process in a half-cell and then removed for use as the anode, matched with LiNi_0.8_Mn_0.1_Co_0.1_O_2_ (NMC811, Canrd Co.,Ltd.) commercial cathode with a fixed areal capacity of 1.7 mAh cm^-2^ (each side). The N/P ratio was chosen to be approximately 1.1. The full cells were subjected to galvanostatic discharge/charge in a voltage range of 2.0-4.25 V. Control samples of homemade graphite and SnS_2_/MoS_2_ electrodes were made using the same preparation process, and assembled in half-cells or full-cells, respectively. Control electrodes of graphite and SnS_2_/MoS_2_ samples were made using the same preparation process and then assembled into half-cells or full-cells, respectively. All the as-assembled cells were allowed to stand for 12 hours to ensure that the electrolyte fully penetrated the electrode before the tests, and at least three cells were used to assess reproducibility.

Cyclic voltammetry (CV) and electrochemical impedance spectroscopy (EIS) were conducted on a Biologic VMP-3 electrochemical workstation over a potential range of 0.01~ 3.0 V (*vs.* Li/Li^+^) and a frequency range of 10^5^ -0.1 Hz with a voltage amplitude of 0.1 mV, respectively. Galvanostatic charge−discharge tests were conducted on a Land CT3001A battery test system. The specific and areal capacities were calculated based on the total mass of the anode material, including the binder and carbon black. Galvanostatic intermittent titration technique (GITT) measurements were conducted under a 10-minute galvanostatic discharge pulse (0.2 A g^-1^), followed by 10 min of relaxation for each pulse. The chemical diffusion coefficient (*D*) can be obtained as:

|  | $D_{{Li}^{+}}=\frac{4}{\pi\tau}\left( \frac{n_{B}V_{m}}{S} \right)^{2}\left( \frac{{\Delta E}_{s}}{{\Delta E}_{\tau}} \right)^{2}$ | (3) |
| --- | --- | --- |

where *τ* is the constant current pulse time (s); $\text{n}_{\text{B}}$ and $V_{m}$ are the mole number (mol) and molar volume (cm^3^ mol^-1^) of the active material, respectively; *S* is the electrode-electrolyte interface area (cm^2^); Δ$\text{E}_{S}$ and Δ$\text{E}_{\tau}$ are the steady-state potential change (V) under the current pulse and the potential change (V) under the constant current pulse after eliminating the *iR* drop, respectively.

***In-situ* XRD patterns measurements:** *In situ* X-ray diffraction (XRD) test was performed to understand the excess Li intercalation−conversion and the reversible electrochemical reaction of the SnS_2_/MoS_2_/DGS electrode. A special half-cell was assembled using a beryllium window for X-ray penetration, where the SnS_2_/MoS_2_/DGS slurry was coated onto the Be window as a working electrode and Li foil was used as a counter electrode. The mass loading of the active materials was 4~5 mg cm^-2^. The cells were cycled at 0.2 A g^-1^ between 0.01 V and 3.0 V (*vs.* Li/Li^+^). *In situ* XRD was determined by a Bruker D8 Advanced diffractometer (Cu-Kα, λ = 1.5406 Å, scan rate of 10° min^-1^), and the obtained XRD patterns were measured at a step scan of 0.2° with scanning every 7~8 minutes in the selected range.

***In situ* thickness evolution monitoring:**  *In situ* thickness measurements were used to determine the volume expansion/contraction of the SnS_2_/MoS_2_/DGS and SnS_2_/MoS_2_ electrodes. The NMC811 cathode, SnS_2_/MoS_2_/DGS anode with the unlithiated thickness, electrolyte, and separator were assembled into a cell mold equipped with a thickness variation sensor. The cell molds were placed in a multichannel expansion analysis system (IEST RSS1400, China) connected to a LAND battery testing system. The corresponding thickness changes during cycling at 0.3 C in a voltage range of 2.0-4.25 V were collected for the first three cycles. All the electrochemical parameters of the cells were tested at room temperature. The expansion ratio (β) can be obtained as:

|  | $\beta=\frac{\Delta\delta}{(\delta_{0}-\delta_{Cu+Al})}\times100\%$ | (4) |
| --- | --- | --- |

Where $\Delta\delta$ is the thickness change value; $\delta_{\text{0}}$ is the electrode thickness value (μm); $\delta_{Cu+Al}$ is the total thickness value of Cu foil and Al foil (μm).


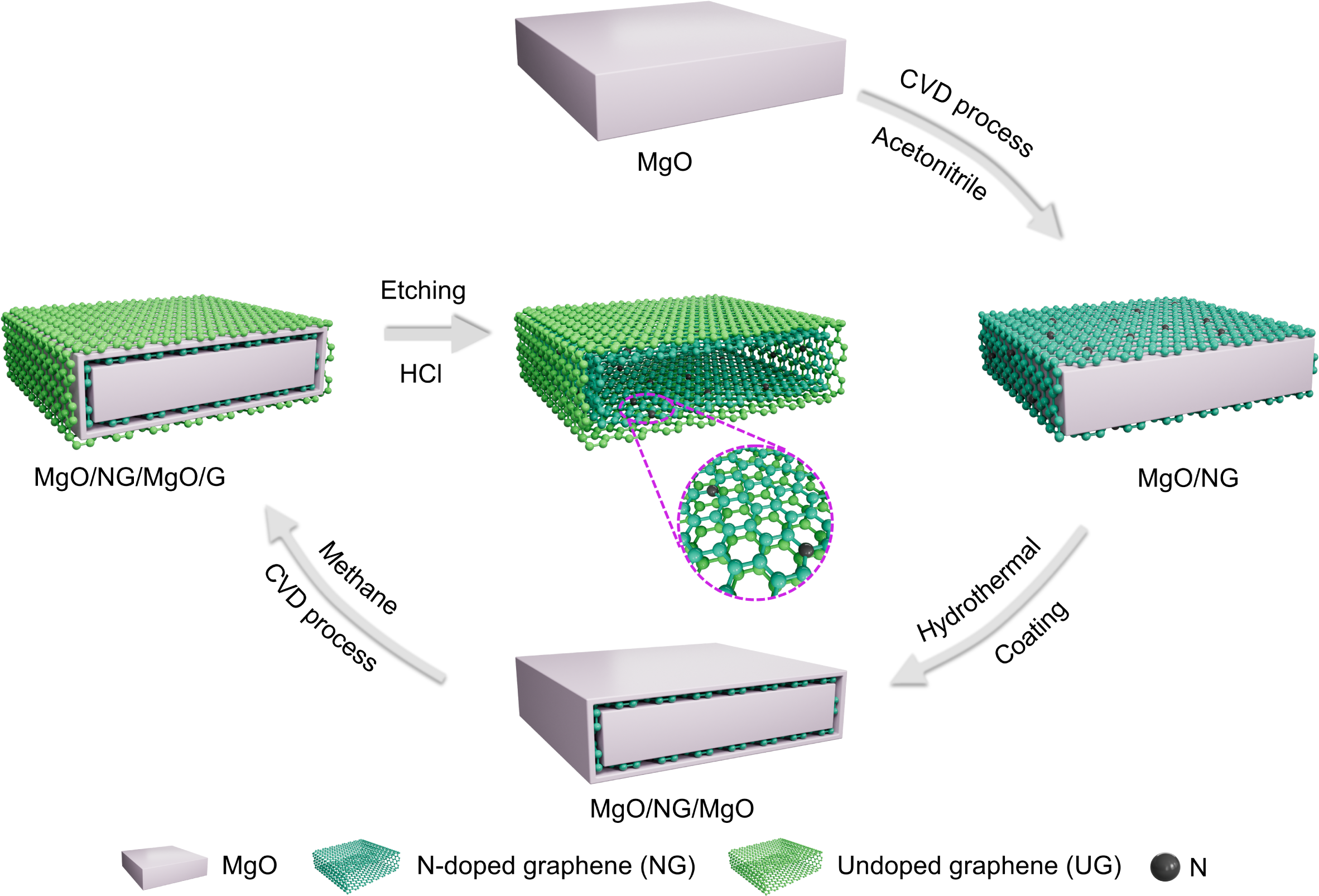


**Figure S1**. Schematic illustration of the synthesis of double-graphene sheet (DGS).

Notes: We first synthesized hydromagnesite (Mg_5_(CO_3_)_4_(OH)) nanosheets as an intermediate template via a facile hydrothermal method,^[1]^ followed by calcination to obtain MgO nanosheets at 450 °C. Nitrogen-doped graphene (NG) was subsequently grown on catalytic MgO substrates by CVD using acetonitrile as the first carbon precursor under an Ar/H_2_ atmosphere at 900 °C. The NG-covered MgO nanosheets were then coated with a thin layer of MgO, onto which graphene was uniformly coated again by CVD using methane as the second carbon source under an Ar atmosphere at 1000 °C. Finally, the MgO template was removed by etching in a 1 M HCl solution, forming a hollow DGS.


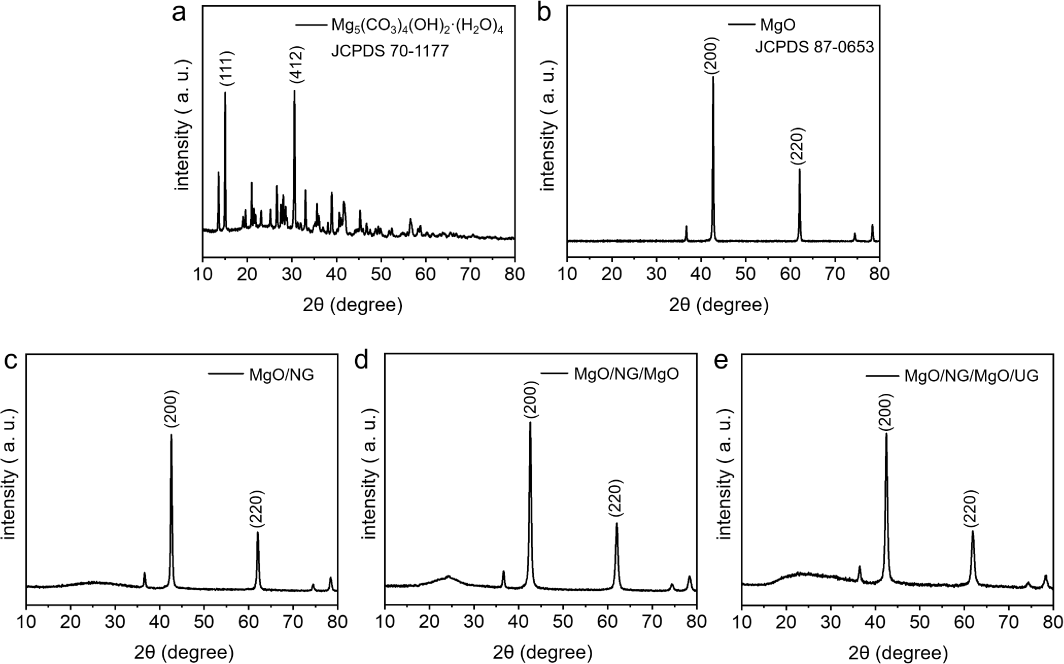


**Figure S2.** XRD patterns of (a) Mg_5_(CO_3_)_4_(OH)_2_·(H_2_O)_4_, (b) MgO, (c) MgO/NG, (d) MgO/NG/MgO, and (e) MgO/NG/MgO/UG.

Notes: The XRD pattern of the as-prepared sample before calcination displays two sharp peaks at 2θ =15.2° and 30.5° assigned to Mg_5_(CO_3_)_4_(OH)_2_ (JCPDS No. 70-1177); the peaks at 2θ =43.0° and 62.5° are assigned to the (200) and (220) reflections of MgO (JCPDS No. 87-0653). The conformal N-doped and undoped graphene layers (denoted as NG and UG) on MgO maintain similar curves, showing a detectable broad peak of approximately 26° ascribed to the carbonaceous structure.^[7]^


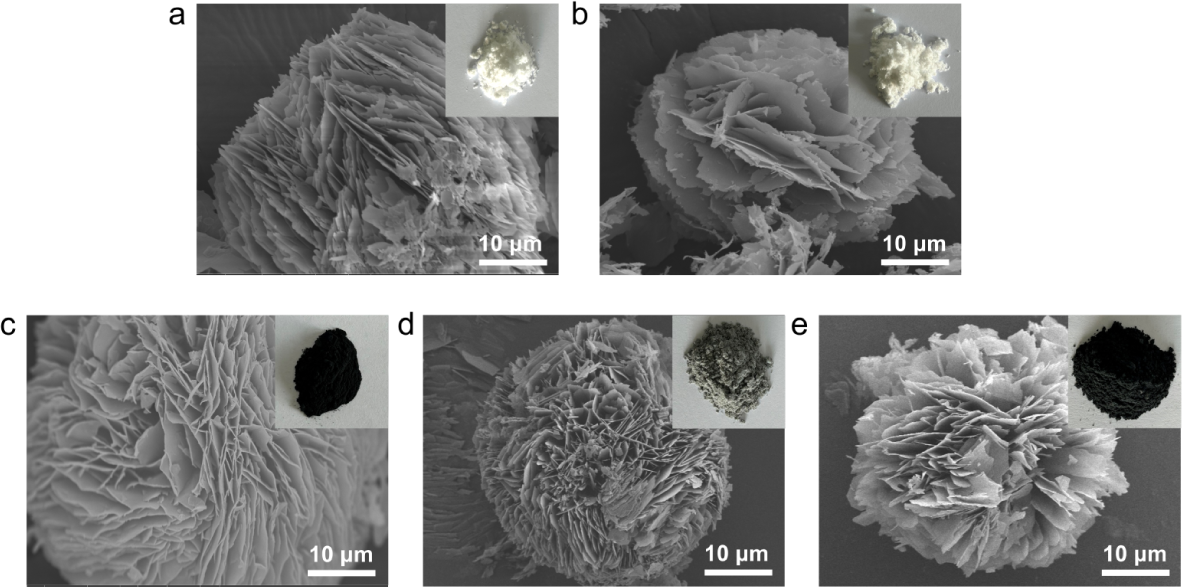


**Figure S3.** SEM images of (a) Mg_5_(CO_3_)_4_(OH)_2_·(H_2_O)_4_, (b) MgO , (c) MgO/NG, (d) MgO/NG/MgO, and (e) MgO/NG/MgO/UG. The insets show corresponding optical images.

Notes: All intermediate samples maintain the morphology of nanoflakes assembled into a flower-like spherical architecture. Acetonitrile and methane CVD turn the white MgO powder into a completely black, indicating that the entire surface of MgO is uniformly coated with an extremely thin carbon layer. With increasing calcination temperature, the nanoflakes exhibit longitudinally growth.^[8]^ Additionally, the size of the nanoflakes increases with increasing calcination temperature up to 900 °C but decreases at 1000 °C, which is consistent with previous reports.^[9]^


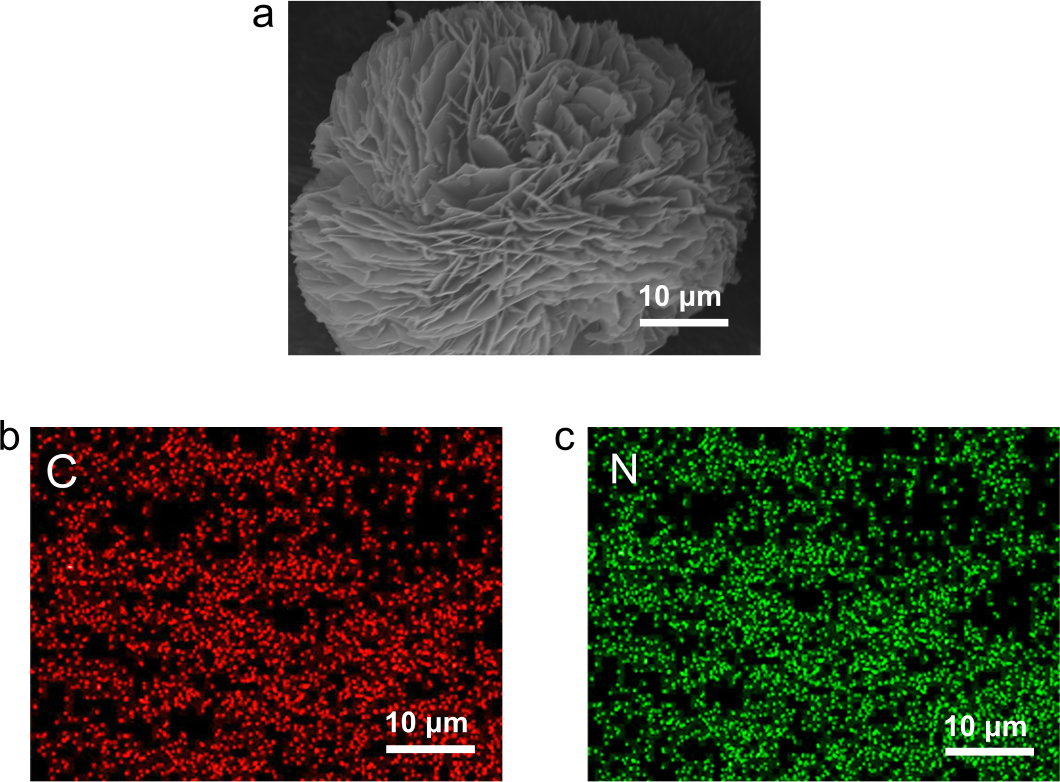


**Figure S4.** (a) SEM-EDS elemental maps of (b) C and (c) N in N-doped graphene.


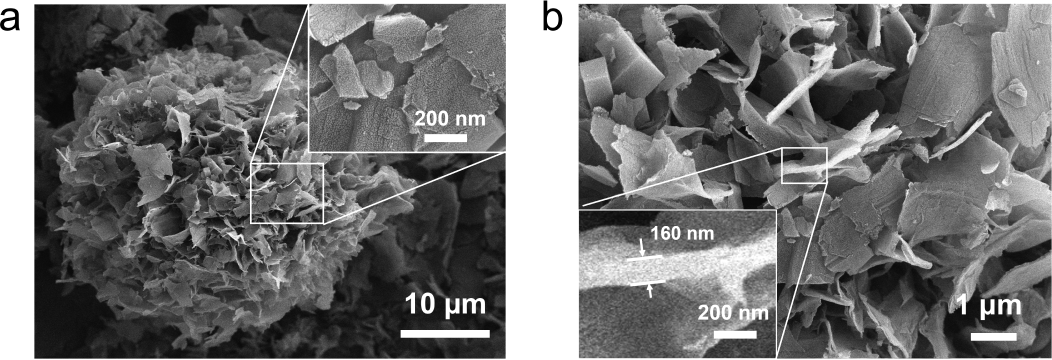


**Figure S5.** SEM images of DGS.


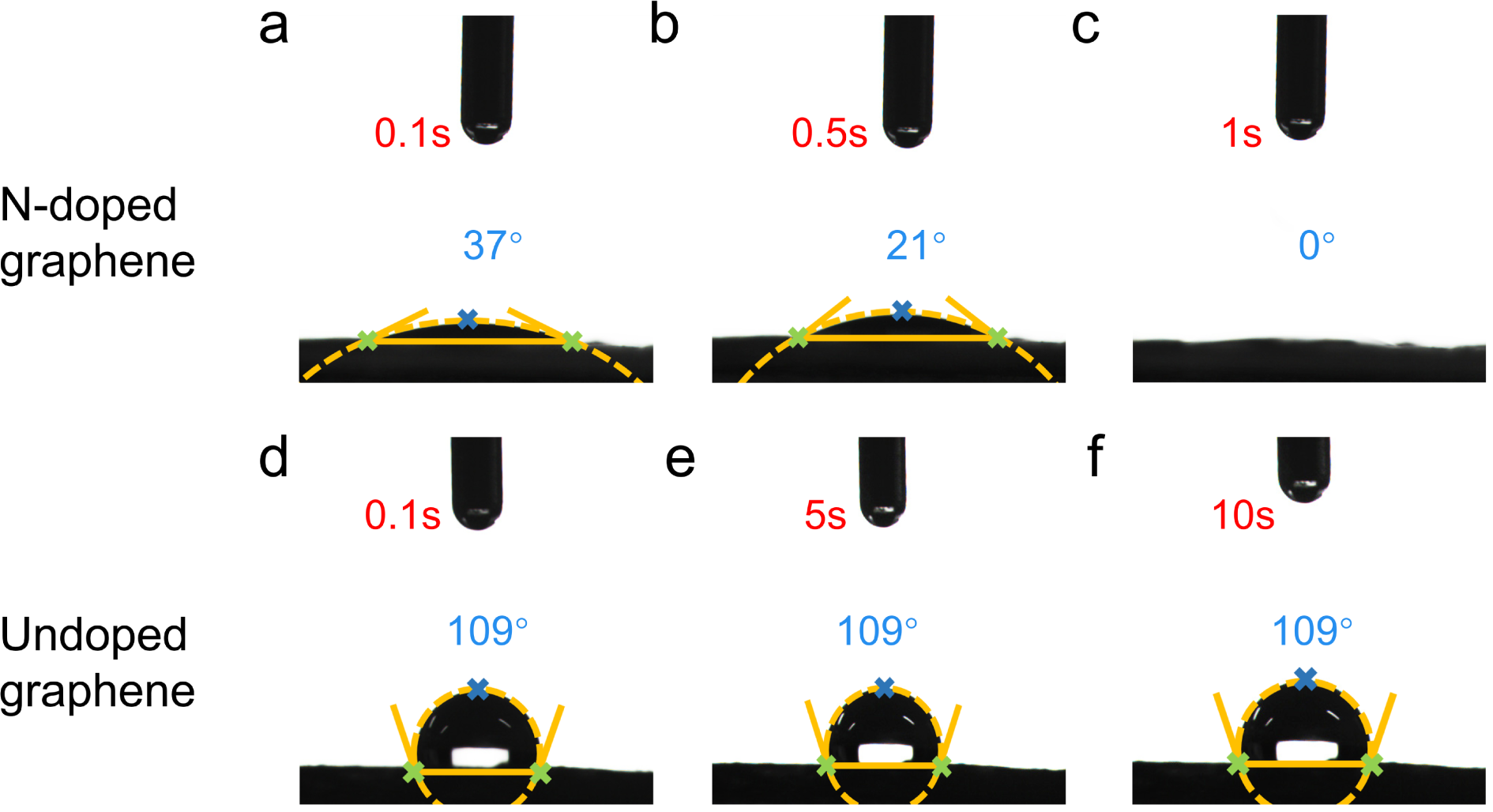


**Figure S6.** The contact angle of water on (a-c) N-doped graphene and (d-f) undoped graphene surface.


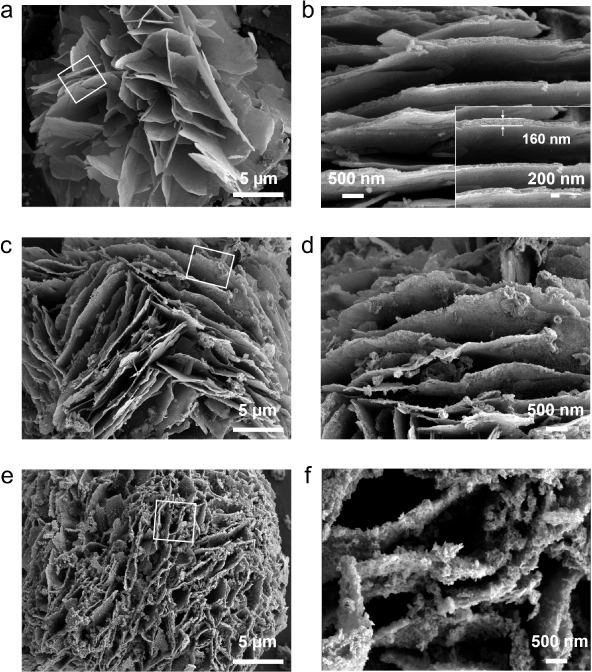


**Figure S7.** SEM images of (a-b) SnS_2_/MoS_2_/DGS, (c-d) MoS_2_/DGS/SnS_2_, and (e-f) SnS_2_/DGS/MoS_2_.


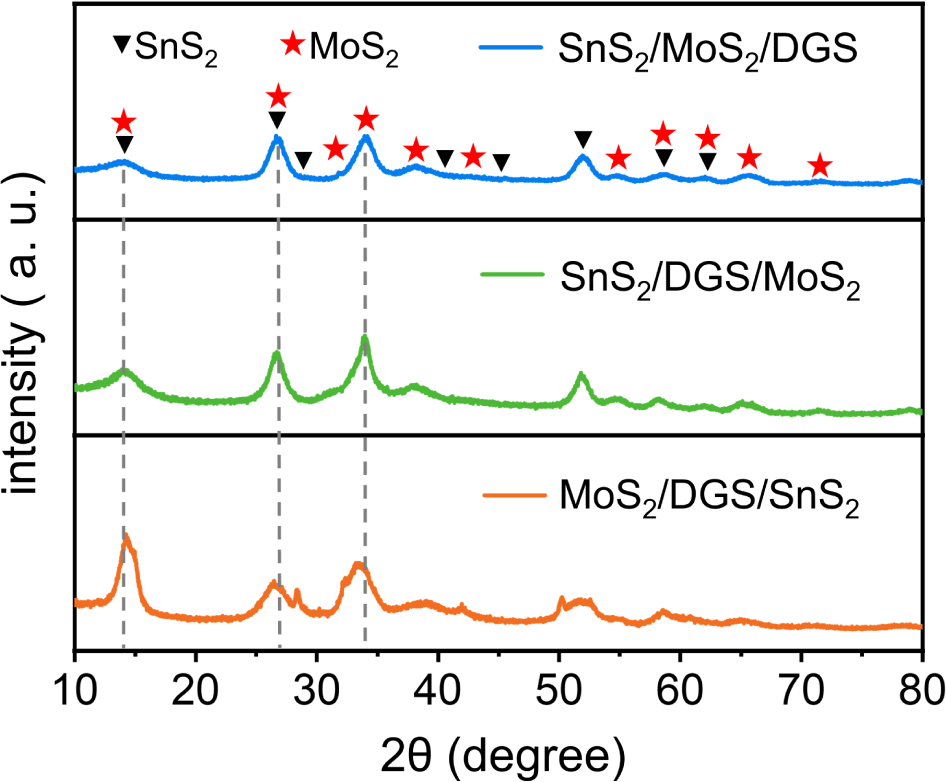


**Figure S8.** (a) XRD patterns of MoS_2_/DGS/SnS_2_, SnS_2_/DGS/MoS_2_, and SnS_2_/MoS_2_/DGS.

Notes: To confirm the experimental procedures in screening the designs, we chose one-step and two-step iterative methods to encapsulate SnS_2_/MoS_2_ into DGS. In the former method, Sn- and Mo-based precursors simultaneously interacted with DGS (SnS_2_/MoS_2_/DGS), while in the latter method, the Sn or Mo precursor was alternatively added to MoS_2_/DGS or SnS_2_/DGS (denoted as MoS_2_/DGS/SnS_2_ or SnS_2_/DGS/MoS_2_). The diffraction peaks of these composites are similar, but the corresponding morphologies of the composites obtained *via* two-step encapsulation show apparent particle agglomeration on the surface and outer edge of DGS, owing to the low ability of the latter precursor to penetrate the inner graphene wrapping sulfide particles ( see Figure S7).


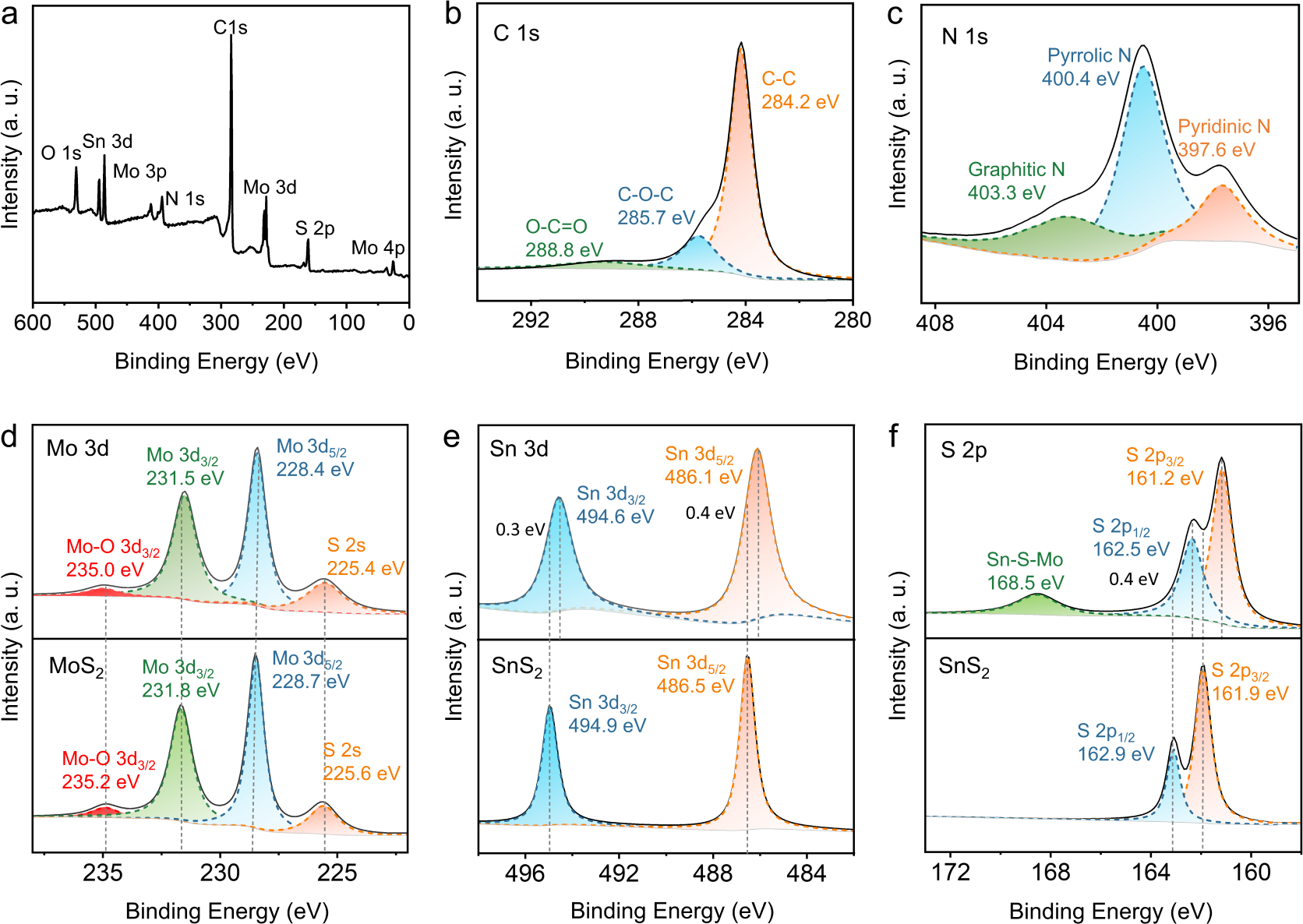


**Figure S9.** (a) XPS survey spectrum of the SnS_2_/MoS_2_/DGS composite, (b) C 1s spectrum, (c) N 1*s* spectrum, (d) Mo 3*d* spectrum, (e) Sn 3*d* spectrum, and (f) S 2*p* spectrum, in comparison with those of pure MoS_2_ and SnS_2_.

**Table S1.** The weight loss of DGS, SnS_2_/MoS_2_, and the SnS_2_/MoS_2_/DGS composite under an air atmosphere at a heating rate of 10 ℃ min^-1^.

| Temperature  [℃] | DGS  [%] | SnS_2_/MoS_2_  [%] | SnS_2_/MoS_2_/DGS  [%] | Process |
| --- | --- | --- | --- | --- |
| 25-200 | 10.5 | 5.3 | 3.8 | Departure of guest water molecules |
| 400-800 | 89.5 | 14.0 | 27.5 | Combustion of DGS, oxidation of TMSs |
| Residues | 0 | 80.7 | 68.7 | SnO_2_ and MoO_3_ |

Notes: The weight loss of the SnS_2_/MoS_2_ composite is divided into two major segments. The first weight loss from 30 ℃ to 200 ℃ represents the loss of guest water molecules. The second weight loss between 200 and 800 ℃ is assigned to two sequential oxidation reactions of MoS_2_ and SnS_2_, yielding final MoO_3_ and SnO_2_ residues of 80.7 wt%, as follows:

|  | | $2MoS_{2}+7O_{2}\to2MoO_{3}+4SO_{2}$ | (4) |
| --- | --- | --- | --- |
|  | $\mathrm{Sn}S_{2}+3O_{2}\to SnO_{2}+2SO_{2}$ | | (5) |

The TGA of DGS shows only one drastic weight loss from 520 to 700 ℃, which is associated with its oxidation reaction. Assuming the complete combustion of DGSs at 700 ℃, the 68.7 wt% residues are attributed to MoO_3_ and SnO_2_, demonstrating that the weight loading of binary sulfides in the composite is 87.5 wt% [DGS wt% in sample = 80.7%-68.7% = 12%, SnS_2_/MoS_2_ wt.% in sample = 100% - 12% - 3.8% = 84.2%, and thus SnS_2_/MoS_2_ wt.% in composites = 84.2%/(84.2% + 12%)= 87.5%].

**Table S2.** ICP − AES results and analysis of the SnS_2_/MoS_2_/DGS composite

| Sample | Sn% | Mo% | Sn/Mo molar ratio |
| --- | --- | --- | --- |
| SnS_2_/MoS_2_/DGS | 40.53 | 8.37 | 4.84 |


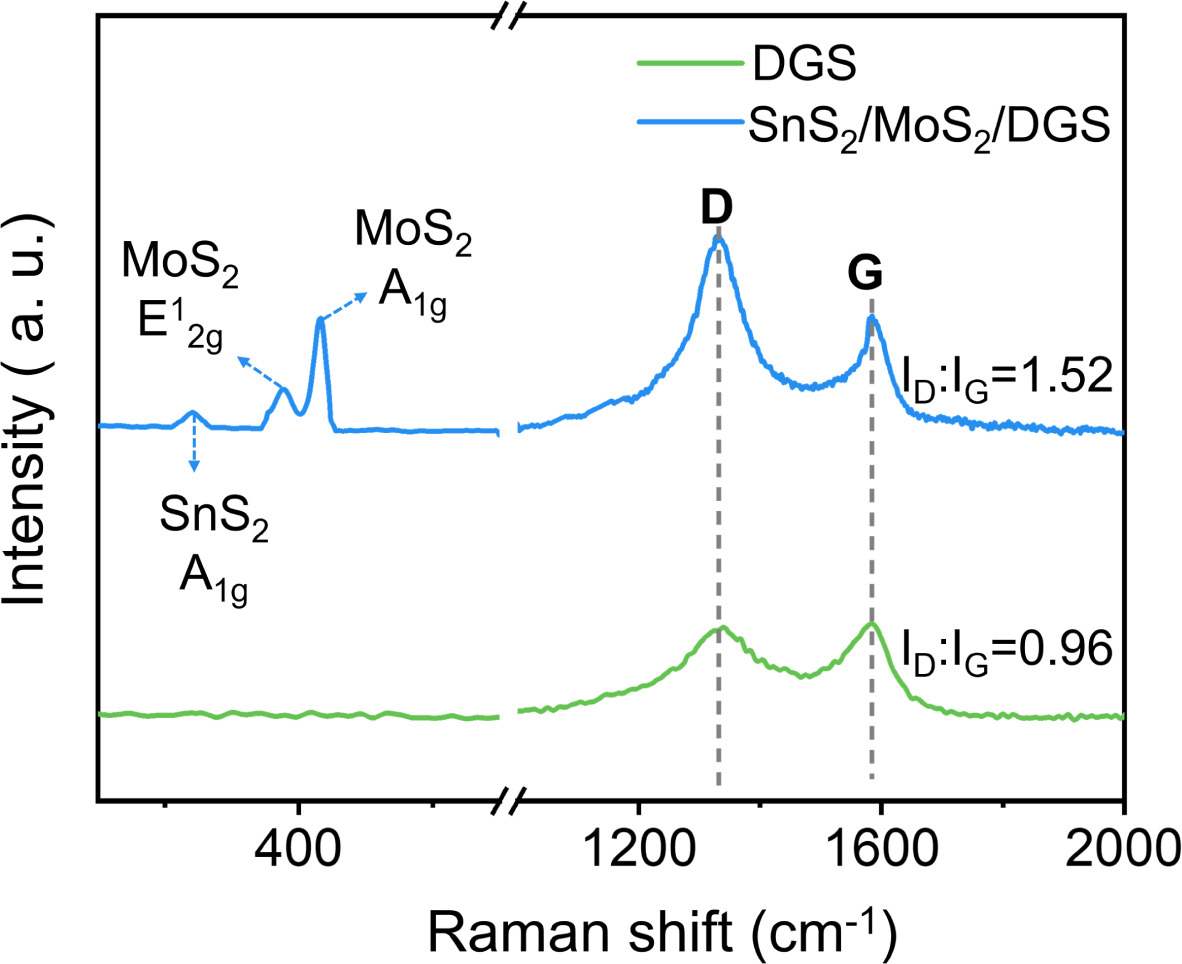


**Figure S10.** Raman spectra of DGS and the SnS_2_/MoS_2_/DGS composite


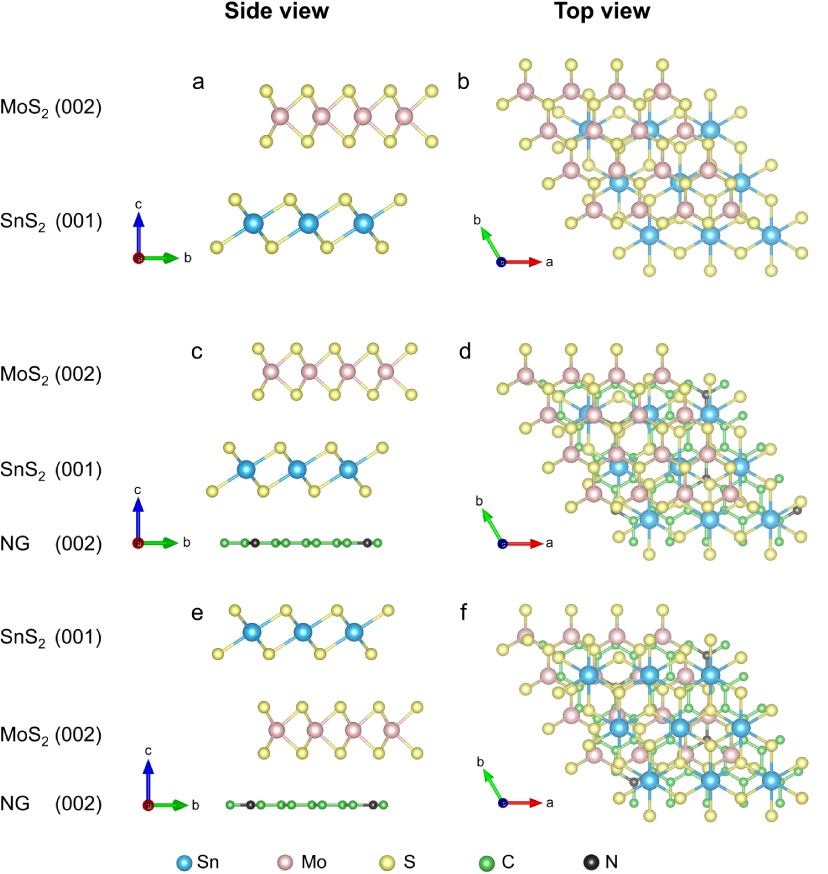


**Figure S11.** Top- and side-views of heterostructure models: (a-b) SnS_2_/MoS_2_, (c-d) MoS_2_/SnS_2_/NG, and (e-f) SnS_2_/MoS_2_/NG.


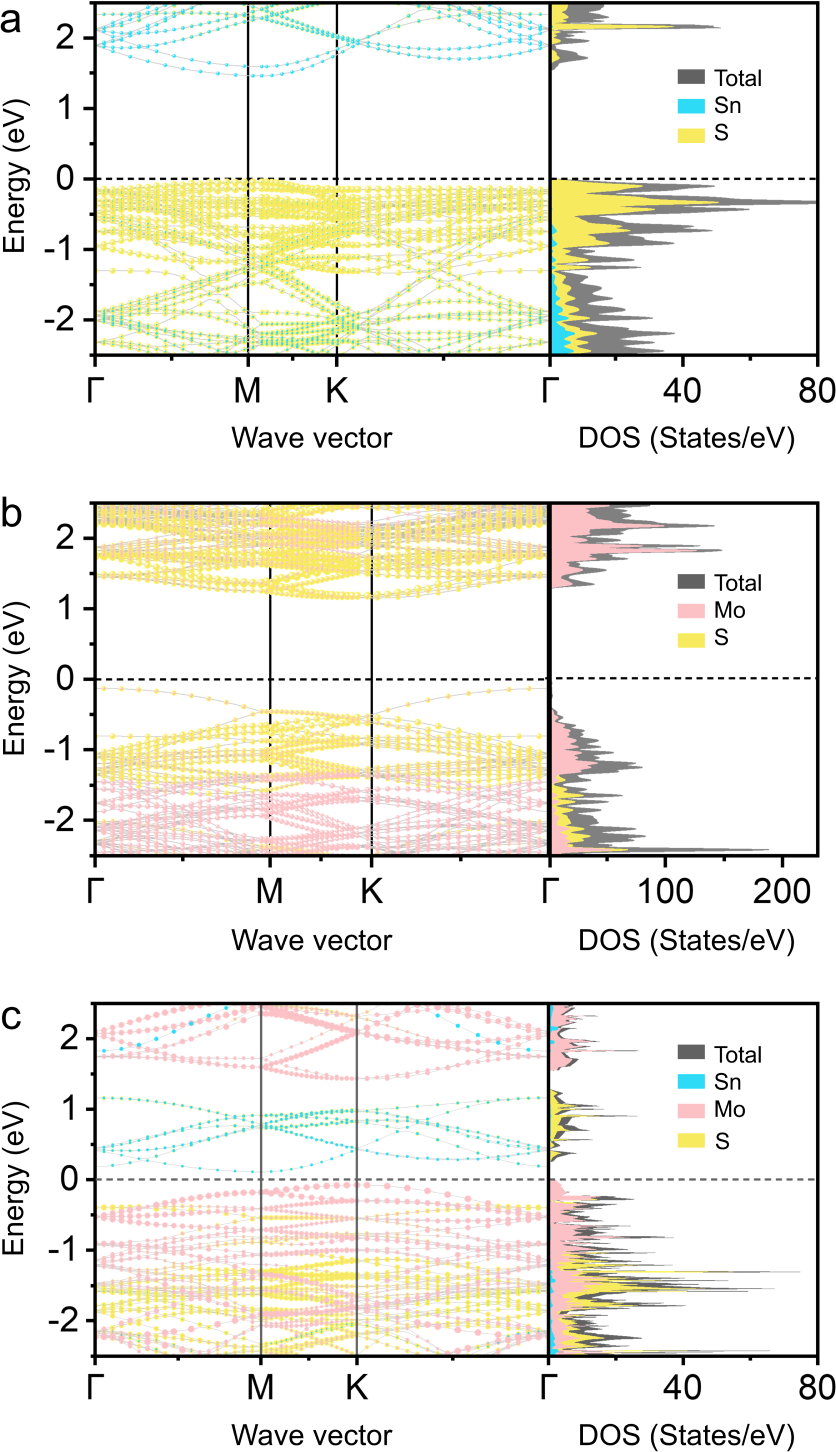


**Figure S12.** Band structures and DOS were obtained through density functional theory calculations: (a) SnS_2_, (b) MoS_2_, and (c) the SnS_2_/MoS_2_ heterojunction.


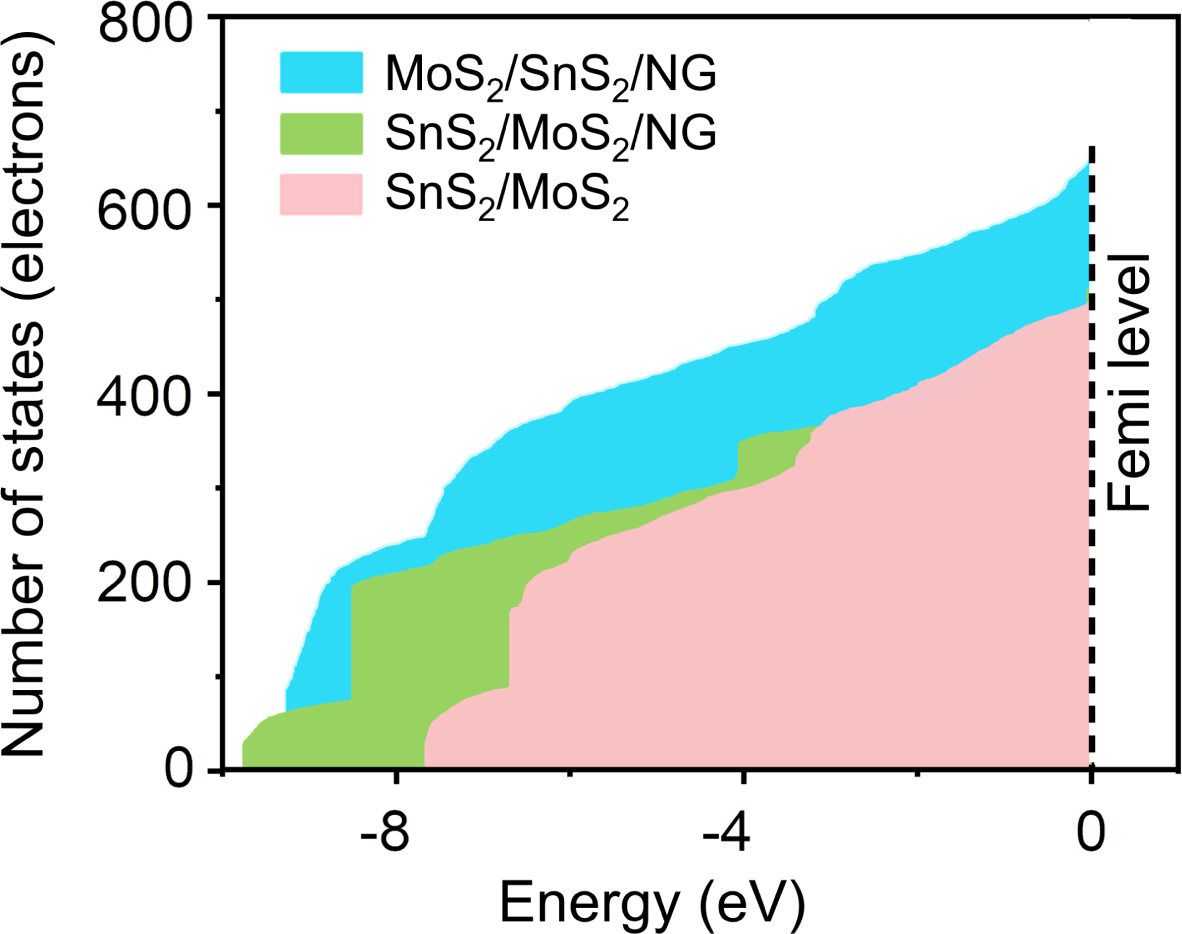


**Figure S13.** Integral DOS (IDOS) of the SnS_2_/MoS_2_, MoS_2_/SnS_2_/NG, and SnS_2_/MoS_2_/NG heterojunctions. The IDOS value represents the total number of electrons at the Fermi level.


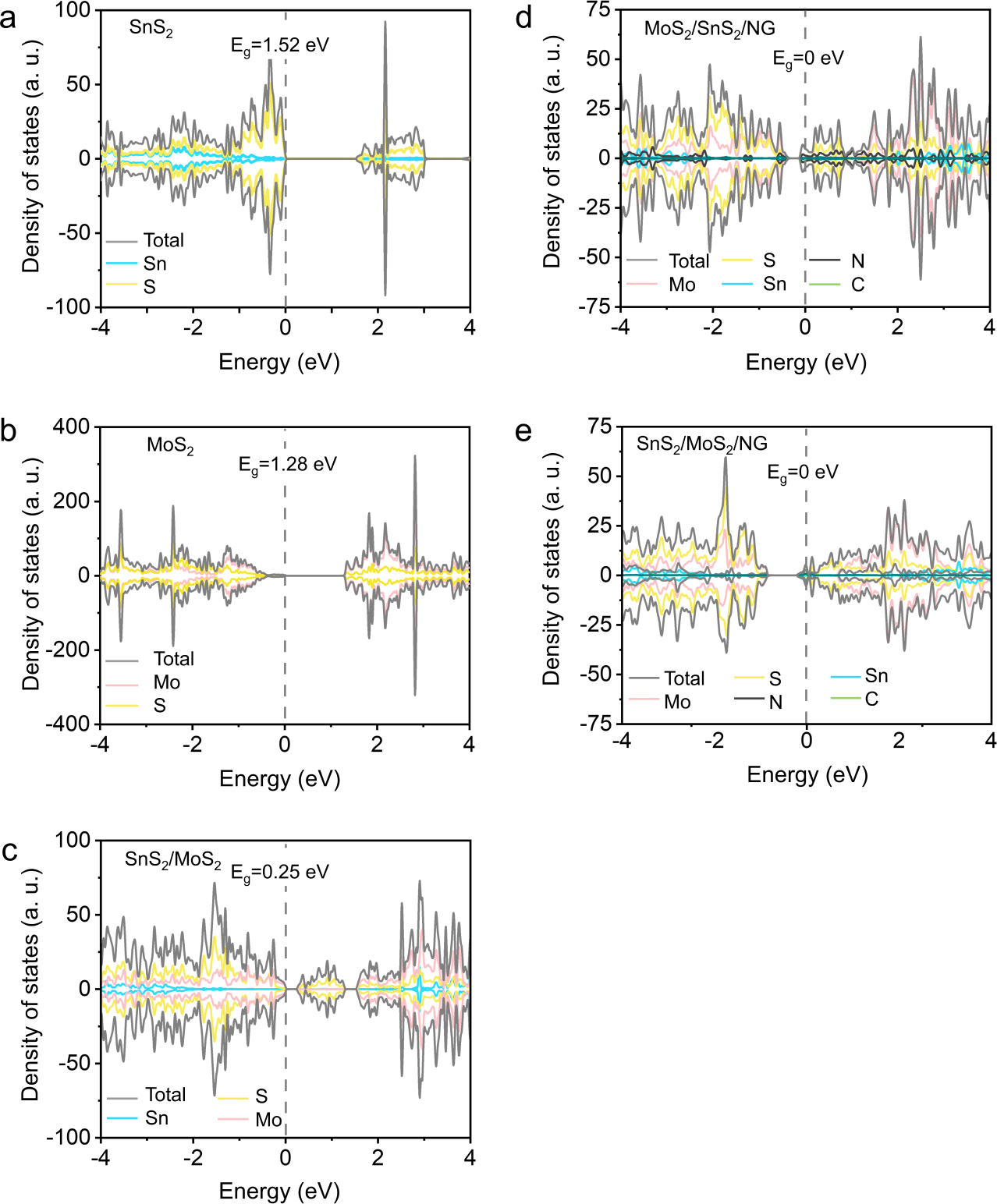


**Figure S14.** Total DOS and partial DOS of (a) SnS_2_, (b) MoS_2_, (c) SnS_2_/MoS_2_, (d) MoS_2_/SnS_2_/NG, and (e) SnS_2_/MoS_2_/NG.


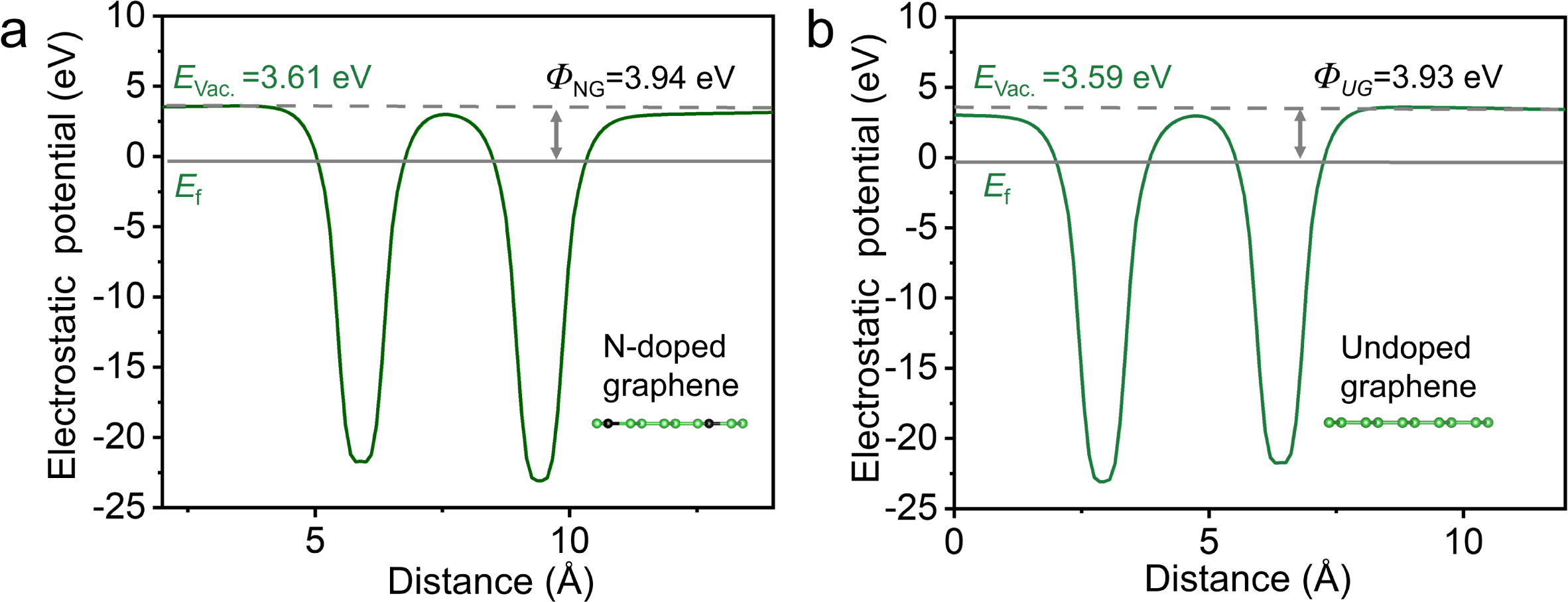


**Figure S15.** Calculated electrostatic potentials for (a) an inner N-doped graphene, and (b) an outer undoped graphene.

Notes: During lithiation, excess lithium ions must be temporarily stored at the interface to maintain the potential difference between the interior and exterior surfaces of a DGS,^[10]^ resulting in storage of excess lithium ions and fast Li^+^ diffusion kinetics.


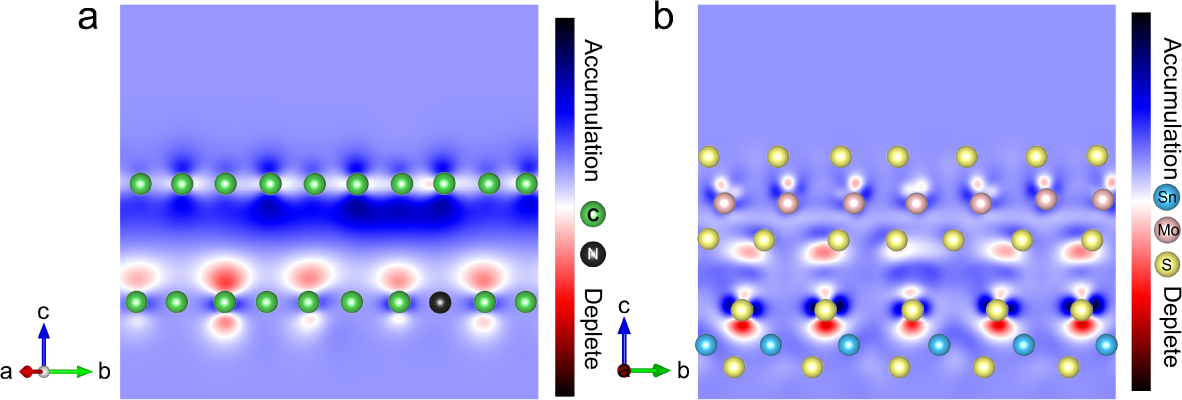


**Figure S16.** Two-dimensional electron density difference distributions of (a) a DGS and (b) the SnS_2_/ MoS_2_ heterojunction. Blue: charge accumulation; Red: charge depletion. The isosurface value is set to 0.0002 e/Bohr^3^.


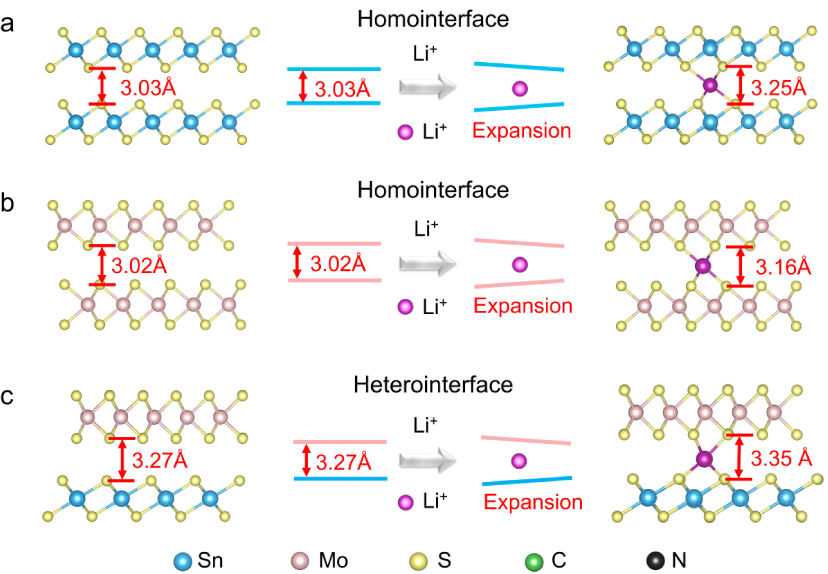


**Figure S17.** Scheme of the calculated atomic structures with the corresponding layer expansion after Li-ion intercalation in (a) SnS_2_, (b) the MoS_2_ homointerface, and (c) the SnS_2_/MoS_2_ heterointerface (Atomic structures and expansion ratio were obtained through density functional theory calculations).


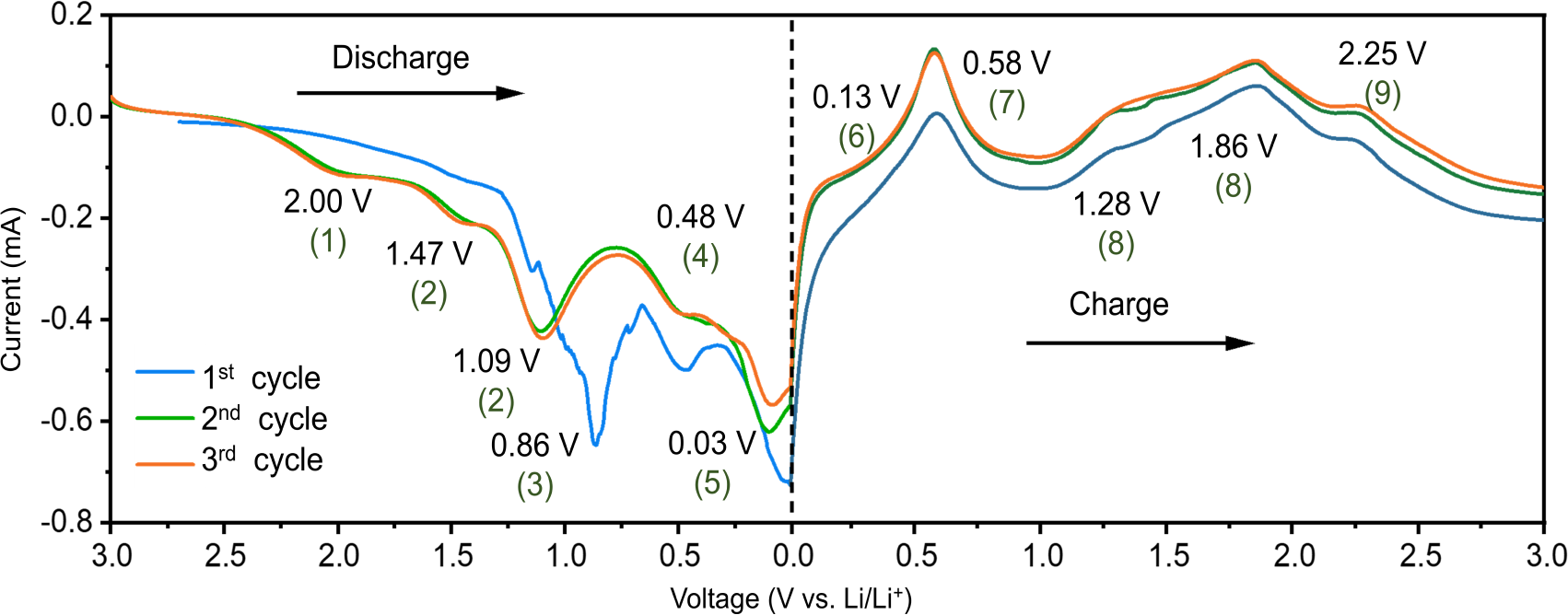


**Figure S18.** CV curves of the first three cycles at a rate of 0.1 mV s^-1^ in a potential range of 0.01 to 3.0 V (*vs*. Li/Li^+^).

**Table S3.** Corresponding redox peak equations.

| Steps | Voltages  (V *vs*. Li^+^/Li) | Reactions | Reaction equations |
| --- | --- | --- | --- |
| (1) | 2.00 | insertion | $\mathrm{SnS}_{2}+x\mathrm{Li}^{+}+xe^{-}\to\mathrm{Li}_{x}\mathrm{SnS}_{2}$  $\mathrm{MoS}_{2}+y\mathrm{Li}^{+}+ye^{-}\to\mathrm{Li}_{y}\mathrm{MoS}_{2}$ |
| (2) | 1.47/1.09 | conversion | $\mathrm{Li}_{x}\mathrm{SnS}_{2}+\left( 4-x \right)Li+\left( 4-x \right)e^{-}\to Sn+\mathrm{Li}_{2}S$  $\mathrm{Li}_{y}\mathrm{MoS}_{2}+\left( 4-y \right)Li+\left( 4-y \right)e^{-}\to Mo+\mathrm{Li}_{2}S$ |
| (3) | 0.86 | \ | $SEI formation$ |
| (4) | 0.48 | alloying | $Sn+4.4\mathrm{Li}^{+}+4.4e^{-}\to\mathrm{Li}_{4.4}\mathrm{Sn}$ |
| (5) | 0.03 | insertion | $6C+z\mathrm{Li}^{+}+ze^{-}\to\mathrm{Li}_{z}C_{6}$ |
| (6) | 0.13 | insertion | $\mathrm{Li}_{z}C_{6}\to6C+z\mathrm{Li}^{+}+ze^{-}$ |
| (7) | 0.58 | dealloying | $\mathrm{Li}_{4.4}Sn\to Sn+4.4\mathrm{Li}^{+}+4.4e^{-}$ |
| (8) | 1.28/1.86 | conversion | $Sn+2\mathrm{Li}_{2}S\to\mathrm{Li}_{x}\mathrm{SnS}_{2}+\left( 4-x \right)Li+\left( 4-x \right)e^{-}$  $Mo+2\mathrm{Li}_{2}S\to\mathrm{Li}_{y}\mathrm{MoS}_{2}+\left( 4-y \right)Li+\left( 4-y \right)e^{-}$ |
| (9) | 2.25 | / | $\mathrm{Li}_{2}S/\mathrm{Li}_{2}S_{2}\to LiS_{n}(4\leq n\leq8)$ |


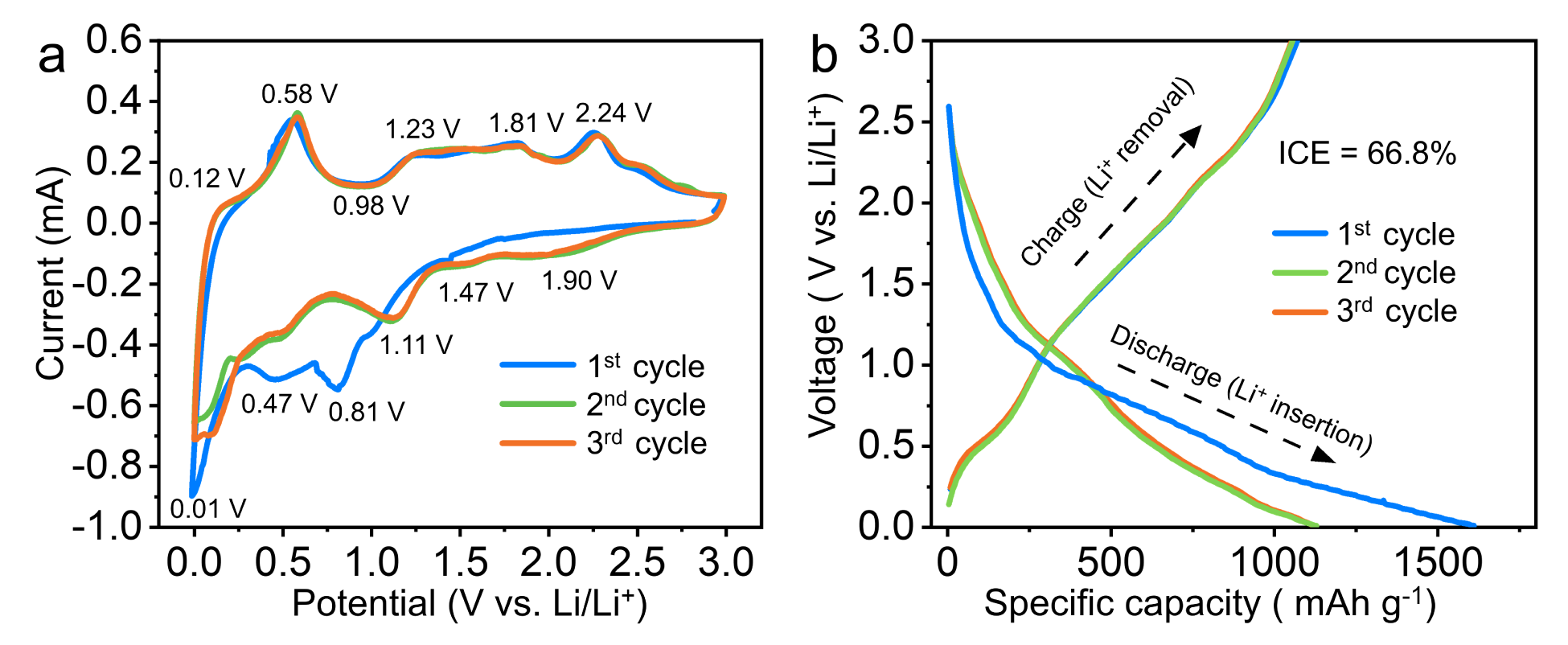


**Figure S19.** (a) CV curves of the SnS_2_/MoS_2_ electrode for the first three cycles at a rate of 0.1 mV s^-1^ in the potential range of 0.01 to 3.0 V (*vs*. Li/Li^+^), and (b) discharge−charge voltage profiles at a current density of 0.2 A g^-1^.


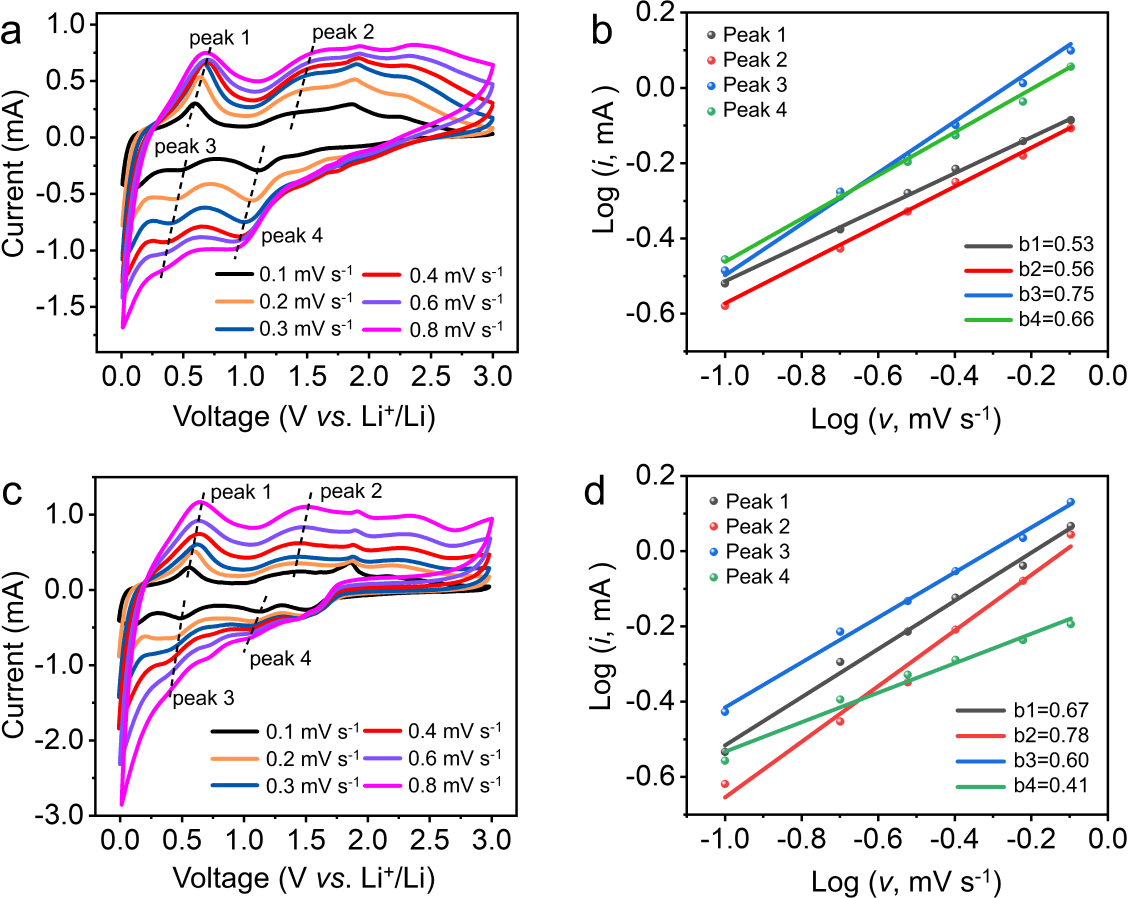


**Figure S20.** CV curves of (a) the SnS_2_/MoS_2_/DGS and (c) SnS_2_/MoS_2_ electrodes at different scan rates from 0.1 to 0.8 mV s^-1^, (b-d) corresponding log (*i*) versus log (*ν*) plots. The resulting *b*−value of each peak was fitted according to *i=aν^b^* (where *i* is the current*, a,* and *b* are varying values, and *ν* is the scanning rate).


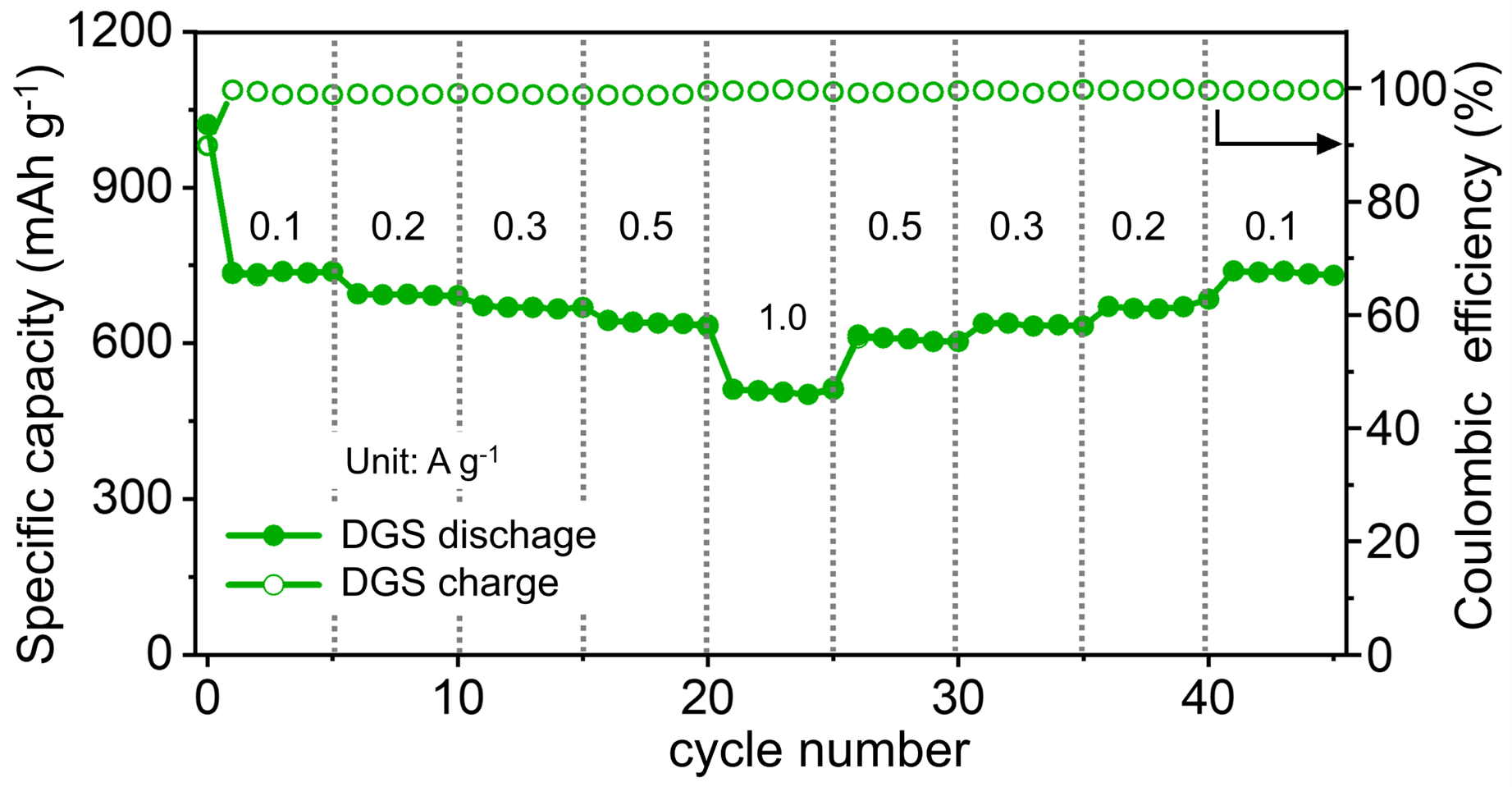


**Figure S21.** The rate capability of the DGS electrode at different current densities**.**


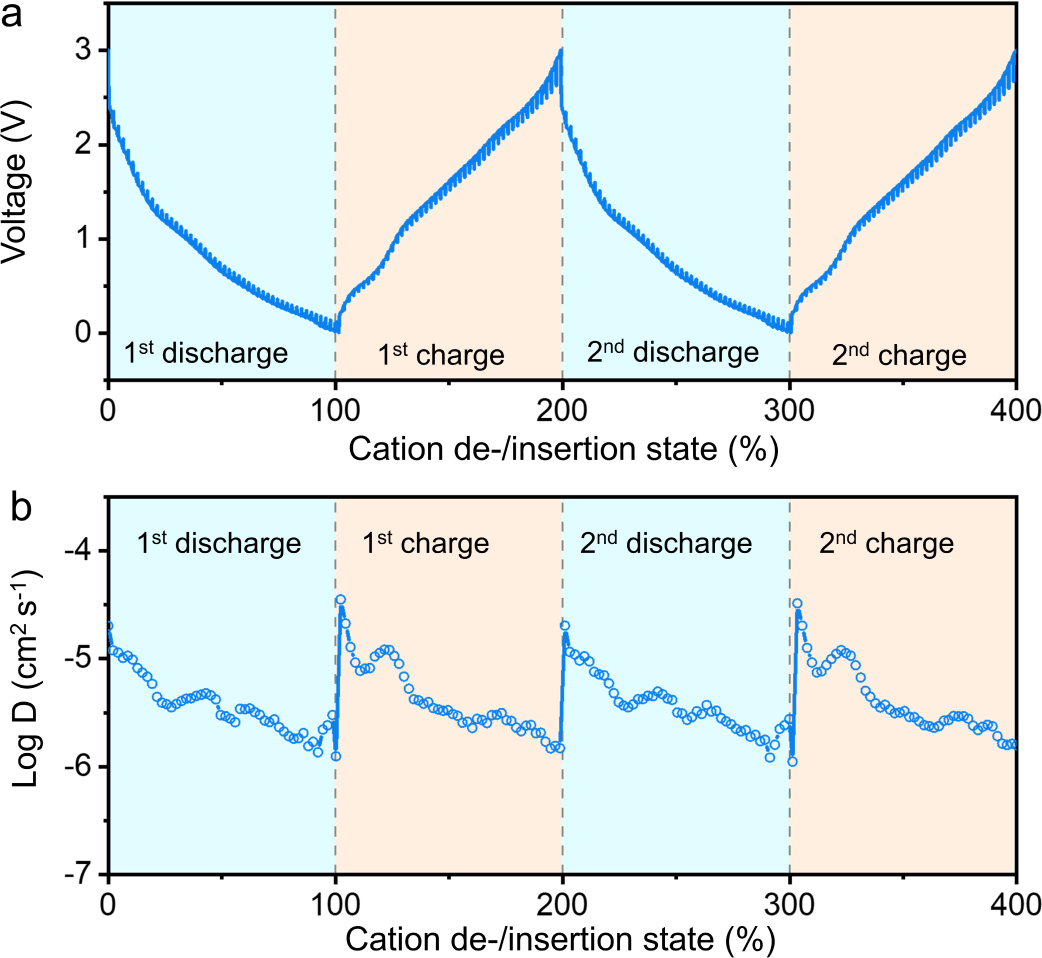


**Figure S22.** GITT profiles of (a) the SnS_2_/MoS_2_/DGS electrodes within two cycles at a current density of 0.2 A g^-1^ with an interval of 10 min and then resting for 10 min in the open-circuit state. (b) Corresponding ion diffusion coefficients ($D_{{Li}^{+}}$).


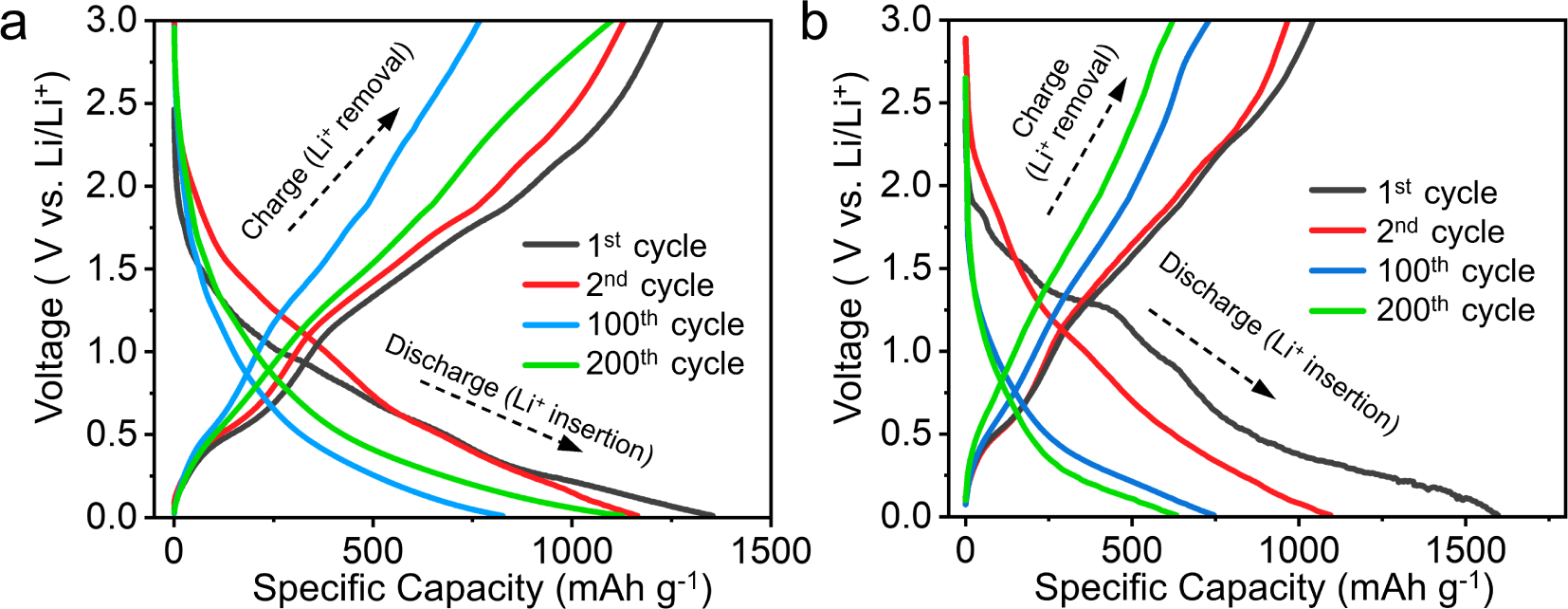


**Figure S23.** Galvanostatic charge−discharge profiles of (a) SnS_2_/MoS_2_/DGS and (b) SnS_2_/MoS_2_ for the 1^st^, 2^nd^, 100^th^ and 200^th^ cycles at a current density of 0.2 A g^−1^.


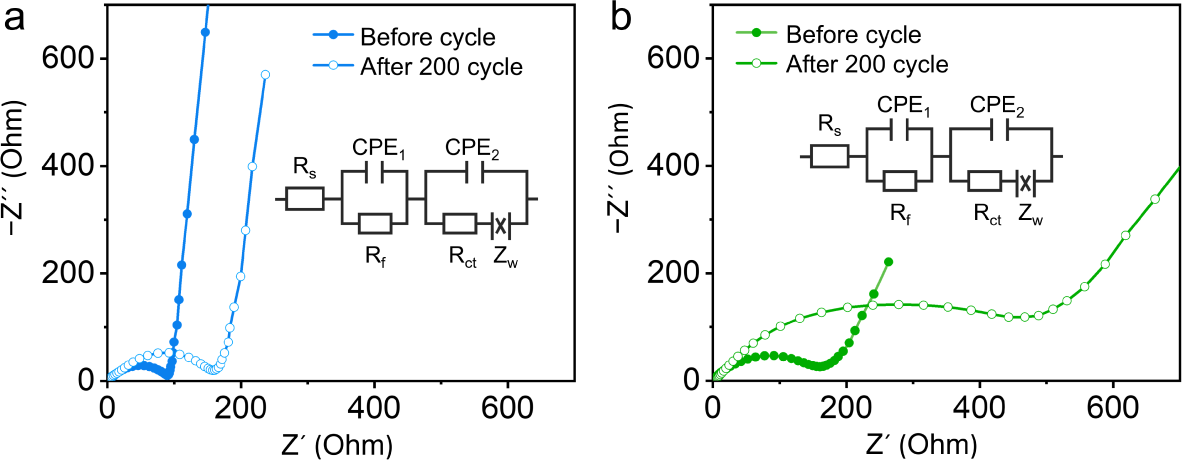


**Figure S24.** Electrochemical impedance spectroscopy (EIS) spectra of (a) SnS_2_/MoS_2_/DGS and (b) SnS_2_/MoS_2_ electrodes at different cycles.

**Table S4.** Resistance by obtained from the equivalent circuit.

|  | Fresh cells | | Cycled cells | |
| --- | --- | --- | --- | --- |
|  | SnS_2_/MoS_2_ | SnS_2_/MoS_2_/DGS | SnS_2_/MoS_2_ | SnS_2_/MoS_2_/DGS |
| R_s_ (Ω) | 9 | 7 | 7 | 4 |
| R_f_ (Ω) | 232 | 108 | 125 | 84 |
| R_ct_ (Ω) | 81 | 49 | 78 | 37 |
| Z_w_ (Ω) | 92 | 152 | 472 | 86 |


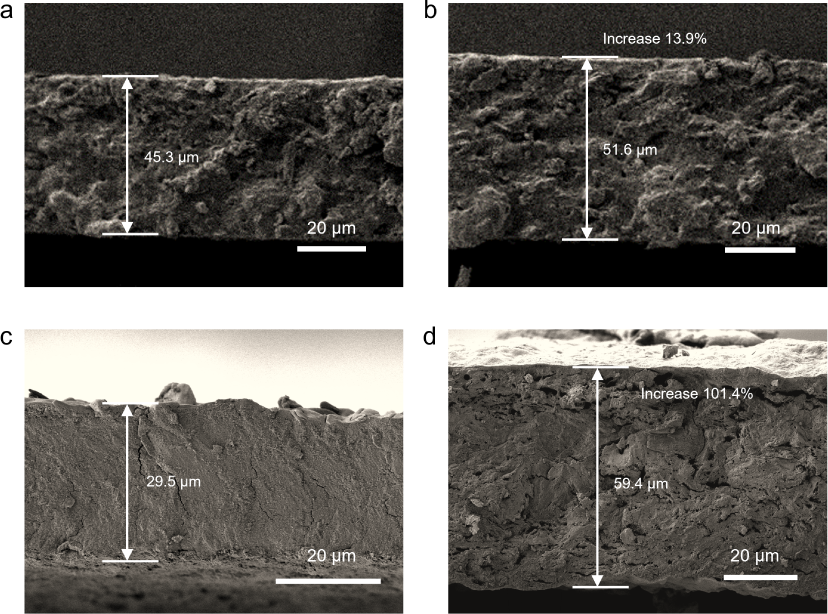


**Figure S25.** Changes in the electrode thickness of the (a-b) SnS_2_/MoS_2_/DGS and (c-d) SnS_2_/MoS_2_ electrodes after 200 cycles at a current density of 0.2 A g^-1^.


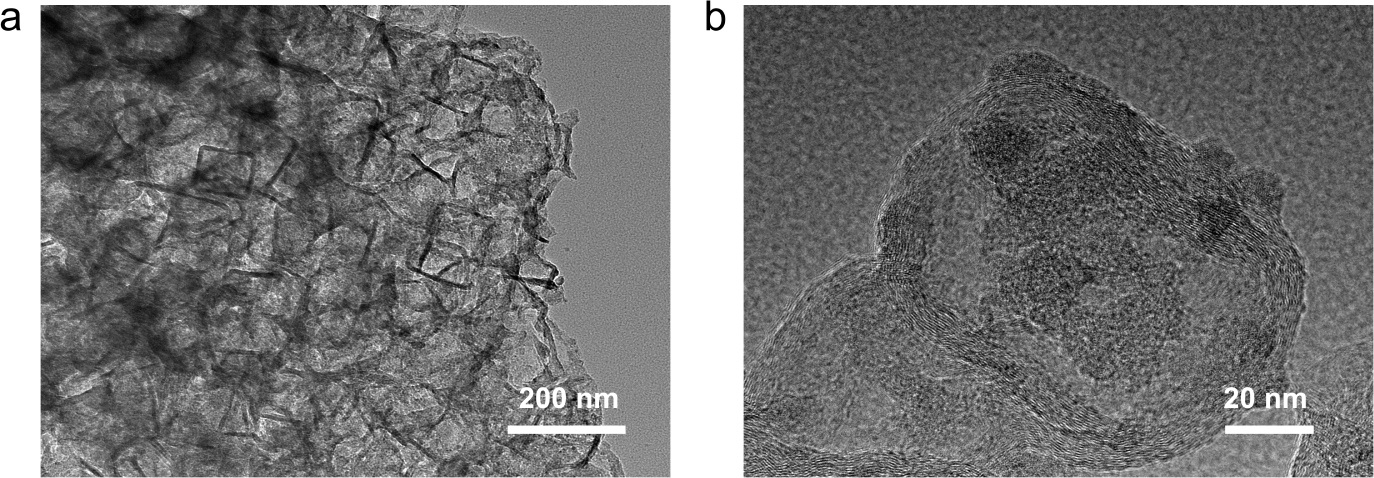


**Figure S26.** TEM images of SnS_2_/MoS_2_/DGS electrodes after 200 cycles at 0.2 A g^-1^


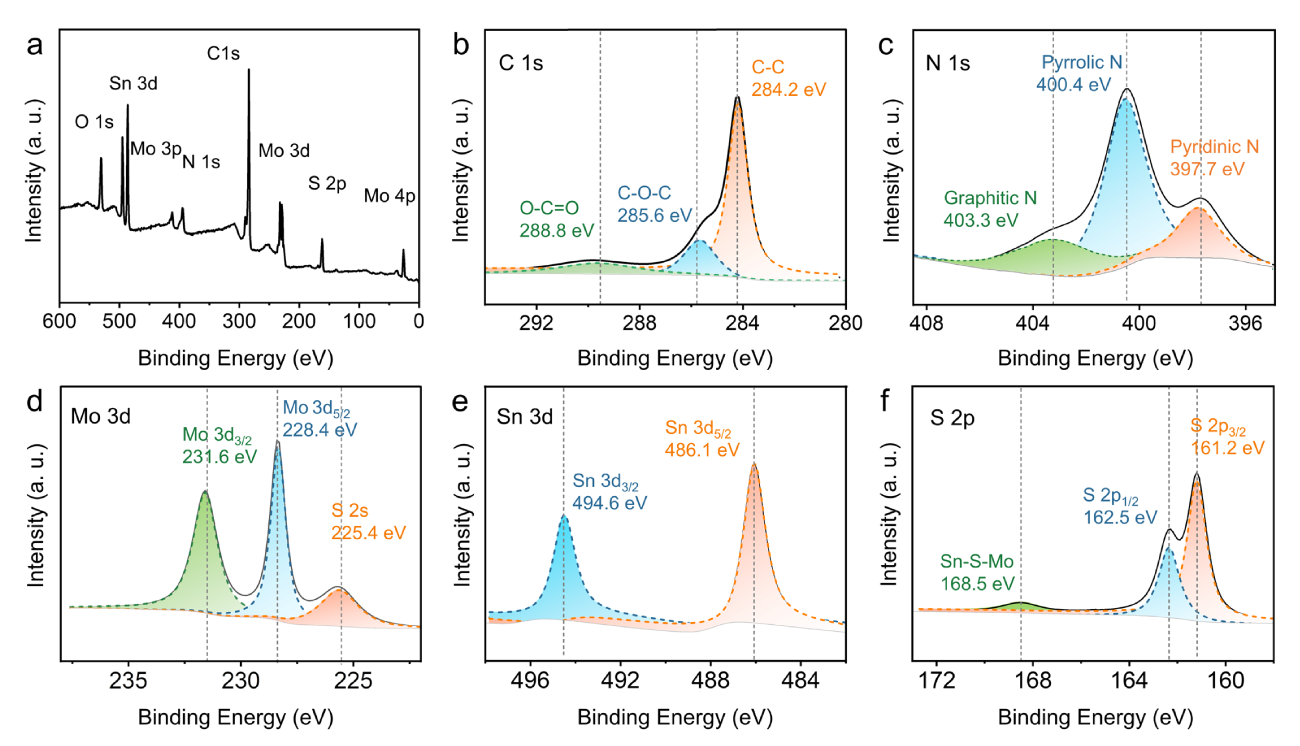


**Figure S27.** (a) XPS survey spectrum of the cycled SnS_2_/MoS_2_/DGS composite, (b) C 1s spectrum, (c) N 1*s* spectrum, (d) Sn 3*d* spectrum, (e) Mo 3*d* spectrum, (f) S 2*p* spectrum.


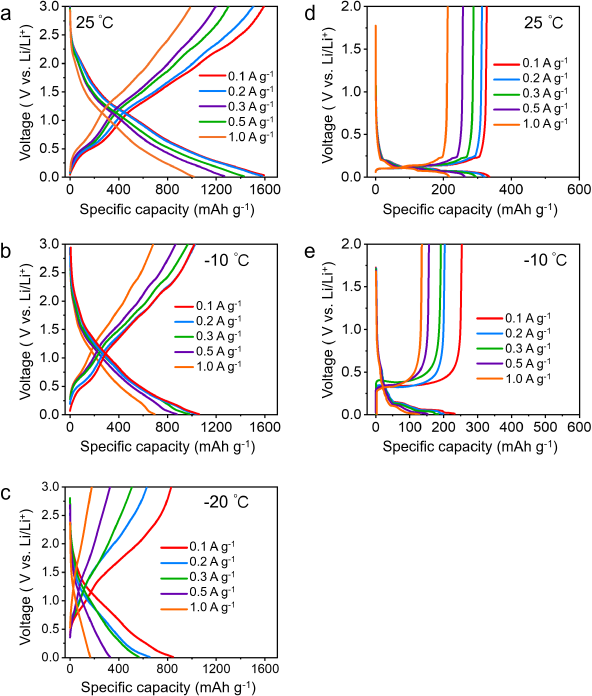


**Figure S28**. Low-temperature operation: the charge/discharge profiles of (a-c) SnS_2_/MoS_2_/DGS electrodes and (d-e) graphite electrodes at different current densities (from 0.1 to 1 A g^-1^) under different temperatures (from 25 to -20 ℃).


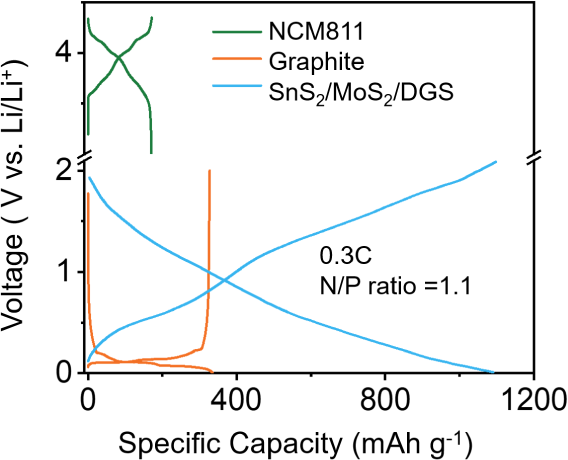


**Figure S29.** Voltage-capacity profiles for NCM811, graphite, and SnS_2_/MoS_2_/DGS electrodes


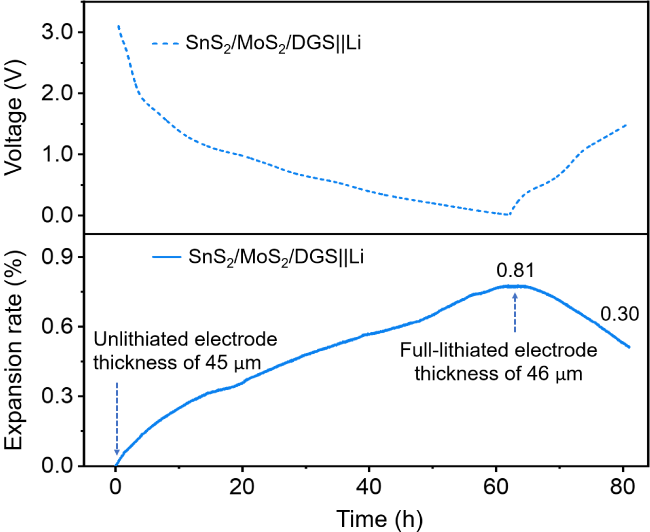


**Figure S30.** Voltage-dependent thickness variation of the SnS_2_/MoS_2_/DGS||Li half-cell during a deep prelithiation down to -0.01 V.


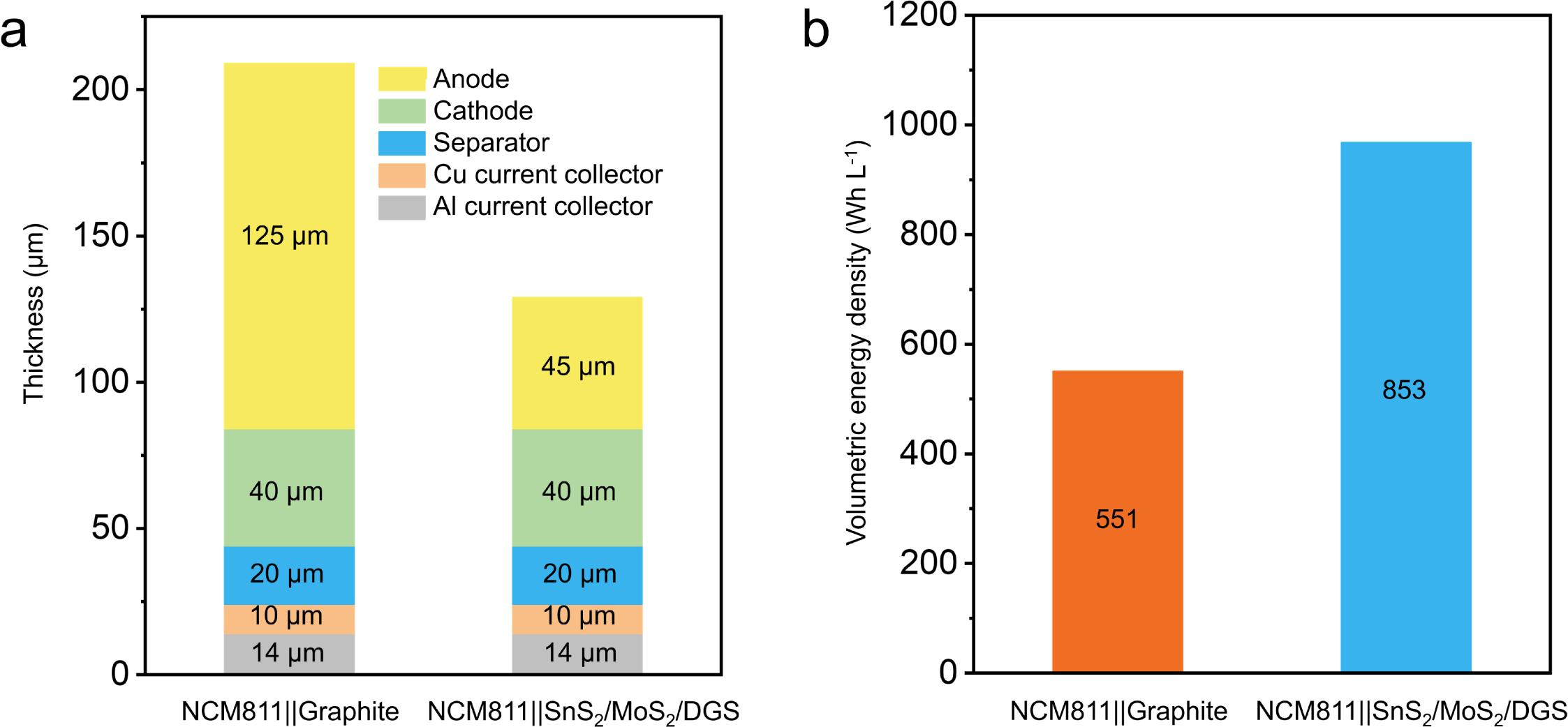


**Figure S31.** (a) Total and each component thicknesses of NCM811||SnS_2_/MoS_2_/DGS and NCM811||graphite full cells. (b) Comparison of volumetric energy densities.

Notes: We only measured the thickness of the one-sided coating on the current collectors, and assumed the thickness of the double-sided coating in the pouch cell. We also assumed that the lean electrolyte was fully absorbed within the electrodes and the separator, for which no extra space was required. The discharge medium voltages are estimated to be 3.2 V and 3.7 V for the NCM811||SnS_2_/MoS_2_/DGS and NCM811||graphite cells, respectively.

**Table S5.** Comparison of the area capacity of SnS_2_/MoS_2_/DGS anodes with those of representative anodes reported at different rates, including anodes consisting of graphite, graphene, Li_4_Ti_5_O_12_, Sn-based composites, heterojunction composites, and Si-based composites.

| Anodes | Voltage  [V *vs*. Li/Li^+^] | Active materials  loading  [mg cm^-2^] | Active materials  mass ratio  [%] | Capacity  [mAh g^-1^] | Areal capacity  [mAh cm^-2^] | Initial Coulombic efficiency  [%] | Ref. |  |
| --- | --- | --- | --- | --- | --- | --- | --- | --- |
| ZnS/Sn | | 0.01-3.0 | 1.3 | 80 | 769  (0.1 A g^-1^) | 0.53  (2.0 mA cm^-2^) | 65.3 | [11] |
| SnSe_2_/SnSe | 0.01-3.0 | 1.2 | 80 | 916  (0.1 A g^-1^) | 0.50  (2.0 mA cm^-2^) | 54.0 | [12] |  |
| Graphite | 0.01-2.0 | 2.2 | 88 | 138  (1.9 A g^-1^) | 0.22  (1.0 mA cm^-2-^) | 88.3 | [13] |  |
| VS_2_/MoS_2_ | 0.01-3.0 | 1.2 | 75 | 1200  (0.5 A g^-1^) | 1.01  (2.0 mA cm^-2^) | 76.0 | [14] |  |
| MoS_2_/GDYO | 0.01-3.0 | 1.5 | 70 | 1267  (0.1 A g^-1^) | 1.11  (2.0 mA cm^-2^) | 59.9 | [15] |  |
| Li_4_Ti_5_O_12_ | 1.0-2.5 | 1.0 | 70 | 130  (1.0 A g^-1^) | 0.22  (2.0 mA cm^-2^) | 94.8 | [16] |  |
| SnS_2−x_P_x_  /RGO | 0.01-3.0 | 1.2 | 80 | 586.4  (5.0 A g^-1^) | 0.75  (2.0 mA cm^-2^) | 60.1 | [17] |  |
| SnS_2_/rGO | 0.05-3.0 | 1.1 | 80 | 722  (0.1 A g^-1^) | 0.44  (2.0 mA cm^-2^) | 58.1 | [18] |  |
| PVA/Si | 0.01-2.0 | 2.0 | 50 | 900  (0.05 A g^-1^) | 2.01  (2.0 mA cm^-2^) | 83.3 | [19] |  |
| SnS_2_/MoS_2_/GS | 0.01-3.0 | 1.3 | 80 | 990  (0.15 A g^-1^) | 0.87  (2.0 mA cm^-2^) | 84.2 | [20] |  |
| MoS_2_/SnS-QDs/CNN | 0.01-3.0 | 1.2 | 80 | 1078  (0.2 A g^-1^) | 0.76  (2.0 mA cm^-2^) | 74.0 | [21] |  |
| SnO_2_/MoS_2_/C | 0.01-3.0 | 1.2 | 80 | 912  (0.2 A g^-1^) | 0.60  (2.0 mA cm^-2^) | 80.0 | [22] |  |
| **SnS_2_/MoS_2_**  **/DGS** | **0.01-3.0** | **6.0** | **80** | **1164**  **(0.2 A g^-1^)** | **2.8**  **(2.0 mA cm^-2^)** | **90.5** | **This work** |  |

**Table S6.** Comparison of the volumetric capacity of SnS_2_/MoS_2_/DGS anodes with those of representative anodes reported in LIBs at different rates

| Anodes | $\rho_{1}$  [g cm^-3]^ | $C_{1}$  [mAh cm^-3^] | $\rho_{2}$  [g cm^-3^] | $C_{2}$  [mAh cm^-3^] | $\rho_{3}$  [g cm^-3^] | $C_{3}$  [mAh cm^-3^] | Current density  [A g^-1^] | Ref. |
| --- | --- | --- | --- | --- | --- | --- | --- | --- |
| Graphite | 1.3 | 526 | NA | NA | NA | NA | 0.2 | [23] |
| C-Li_4_Ti_5_O_12_ | 1.1 | 195 | NA | NA | NA | NA | 0.2 | [24] |
| SnS_2_/rGO | 1.9 | 1087 | NA | NA | NA | NA | 0.2 | [25] |
| PVP-Sn  /Ti_3_C_2_ | 2.1 | 1375 | NA | NA | NA | NA | 100 | [26] |
| HD-Si  /Ti_3_C_2_T_x_  /G | 2.0 | 2160 | NA | NA | NA | NA | 1.0 | [27] |
| Porous Si | 1.0 | 1697 | NA | NA | NA | NA | 0.17 | [28] |
| SnO_2_  /3D CNT | NA | 1700 | NA | NA | NA | NA | 2.1 | [29] |
| **SnS_2_/MoS_2_**  **/DGS** | **2.2** | **2420** | **1.2** | **1320** | **1.1** | **1210** | **0.2** | **This work** |

Notes: The areal capacities were calculated by the total mass of electrode materials. In Table S6, $\rho_{1}$ is the density of active material (g cm^-3^); $C_{1}$ is the volumetric capacity based on the active material (mAh cm^-3^); $\rho_{2}$ is the density of electrode; $C_{2}$ is the volumetric capacity based on the unlithiated electrode (mAh cm^-3^); $\rho_{3}$ is the density of full-lithiated electrode (g cm^-3^); $C_{3}$ is the volumetric capacity based on the full-lithiated electrode (mAh cm^-3^), respectively.

**Table S7**. Typical parameters to calculate the gravimetric and volumetric energy densities

| Components | Parameters | Unit | Value |
| --- | --- | --- | --- |
| NCM811  cathode | Discharge capacity | mAh g^-1^ | 190 |
|  | Voltage window | V *vs*. Li/Li^+^ | 3~4.3 |
|  | Active material loading | \ | 95% |
|  | Area weight (each side) | mg cm^-2^ | 9.5 |
|  | Areal capacity (each side) | mAh cm^-2^ | 1.7 |
|  | Electrode thickness (each side) | μm | 20.0 |
|  | Al foil thickness | μm | 14.0 |
| SnS_2_/MoS_2_/DGS  anode | Discharge capacity | mAh g^-1^ | 1100 |
|  | Voltage window | V *vs*. Li/Li^+^ | 0.01~2.0 |
|  | Active material loading | % | 80 |
|  | Area weight (each side) | mg cm^-2^ | ~2.0 |
|  | Areal capacity (each side) | mAh cm^-2^ | 1.86 |
|  | Electrode thickness (each side) | μm | 22.5 |
|  | Cu foil thickness | μm | 10.0 |
|  | N/P | \ | 1.1 |
| Electrolyte | electrolyte/capacity | g Ah^-1^ | 3.0 |
| Separator | Thickness | μm | 20 |
| Coin cell | Voltage | V | 3.2 |
|  | Capacity | mAh | 3.86 |
|  | Volume | μL | 14.52 |
|  | Mass | mg | 56.22 |
|  | Gravimetric energy density | Wh kg^-1^ | 577 |
|  | Volumetric energy density | Wh L^-1^ | 853 |

Notes: the energy densities were calculated by the total mass of cells (excluding shells of coin cells).

**References**

[1] Z. Jin, Y. Jia, K.-S. Zhang, L.-T. Kong, B. Sun, W. Shen, F.-L. Meng, J.-H. Liu, *J. Alloys Compd.* **2016**, *675*, 292.

[2] S. Grimme, J. Antony, S. Ehrlich, H. Krieg, *J. Chem. Phys.* **2010**, *132*, 154104.

[3] G. Kresse, D. Joubert, *Phys. Rev. B* **1999**, *59*, 1758.

[4] G. Cai, Z. Cao, F. Xie, H. Jia, W. Liu, Y. Wang, F. Liu, X. Ren, S. Meng, M. Liu, *Materials Futures* **2024**, *3*, 025601.

[5] J. Cheng, Z. Niu, Z. Zhao, X. Pei, S. Zhang, H. Wang, D. Li, Z. Guo, *Adv. Energy Mater.* **2022**, *13*, 202203248.

[6] E. E. Fesenko, L. M. Mezhevikina, M. A. Osipenko, R. Y. Gordon, S. S. Khutzian, *Electromagn. Biol. Med.* **2010**, *29*, 1.

[7] J. P. Dhal, M. Sethi, B. G. Mishra, G. Hota, *Mater. Lett.* **2015**, *141*, 267.

[8] S. K K, A. Saji, A. Chanda, M. Vasundhara, *Opt. Mater.* **2022**, *132*, 112777.

[9] A. Hazra Chowdhury, I. Hazra Chowdhury, M. Kanti Naskar, *Mater. Lett.* **2015**, *158*, 190.

[10] W. J. Yu, L. Zhang, P. X. Hou, F. Li, C. Liu, H. M. Cheng, *Adv. Energy Mater.* **2015**, *6*, 1501755.

[11] W. Feng, X. Wen, Y. Wang, L. Song, X. Li, R. Du, J. Yang, H. Li, J. He, J. Shi, *Adv. Sci.* **2023**, *10*, e2204671.

[12] C. Ke, R. Shao, Y. Zhang, Z. Sun, S. Qi, H. Zhang, M. Li, Z. Chen, Y. Wang, B. Sa, H. Lin, H. Liu, M. S. Wang, S. Chen, Q. Zhang, *Adv. Funct. Mater.* **2022**, *32*, 2205635.

[13] H. Wang, N. Qin, Y. Li, Z. Li, F. Zhang, W. Luo, C. Zeng, Z. Lu, H. Cheng, *Carbon* **2023**, *205*, 435.

[14] Y. Dong, Y. Liu, Y. Hu, K. Ma, H. Jiang, C. Li, *Sci. Bull.* **2020**, *65*, 1470.

[15] T. Wang, M. Li, L. Qi, P. Jie, W. Yang, Y. Li, *Adv. Funct. Mater.* **2023**, *33*, 202308470.

[16] Y. Zhang, J. Huang, N. Saito, X. Yang, Z. Zhang, L. Yang, S. i. Hirano, *Adv. Energy Mater.* **2022**, *12*, 202200922.

[17] Z. Kong, M. Huang, Z. Liang, H. Tu, K. Zhang, Y. Shao, Y. Wu, X. Hao, *Inorg. Chem. Front.* **2022**, *9*, 902.

[18] M. Cheng, Q. Hu, C. Du, J. Li, W. Liao, J. Li, X. Huang, *J. Solid State Chem.* **2021**, *296*, 122022.

[19] Y. Pei, Y. Wang, A.-Y. Chang, Y. Liao, S. Zhang, X. Wen, S. Wang, *Carbon* **2023**, *203*, 436.

[20] Y. Jiang, Y. Guo, W. Lu, Z. Feng, B. Xi, S. Kai, J. Zhang, J. Feng and S. Xiong, *ACS Appl. Mater. Interfaces*, **2017**, 9, 27697-27706.

[21] G. Ke, H. Chen, J. He, X. Wu, Y. Gao, Y. Li, H. Mi, Q. Zhang, C. He and X. Ren, *Chem. Eng. J.*, **2021**, 403, 126251.

[22] P. Mao, Y. Wang, W. Guo, W. Zhang, T. He, S. Dong, P. Xiao and S. Rao, *J. Alloys Compd.*, **2021**, 850, 156745.

[23] M. Wang, J. Wang, J. Xiao, N. Ren, B. Pan, C. S. Chen, C. H. Chen, *ACS Appl. Mater. Interfaces* **2022**, *14*, 16279.

[24] H.-G. Jung, J. Kim, B. Scrosati, Y.-K. Sun, *J. Power Sources* **2016**, *196*, 7763.

[25] F. Xin, M. S. Whittingham, *Electrochem. Energy Rev.* **2020**, *3*, 643.

[26] J. Luo, X. Tao, J. Zhang, Y. Xia, H. Huang, L. Zhang, Y. Gan, C. Liang, W. Zhang, *ACS Nano* **2016**, *10*, 2491.

[27] Z. Liu, D. Lu, W. Wang, L. Yue, J. Zhu, L. Zhao, H. Zheng, J. Wang, Y. Li, *ACS Nano* **2022**, *16*, 4642.

[28] L. Cao, T. Huang, Q. Zhang, M. Cui, J. Xu, R. Xiao, *ACS Appl. Mater. Interfaces* **2020**, *12*, 57071.

[29] J. Liu, X. Chen, J. Kim, Q. Zheng, H. Ning, P. Sun, X. Huang, J. Liu, J. Niu, P. V. Braun, *Nano Lett.* **2016**, *16*, 4501.
